# Effectiveness of interventions on conscience: Findings of a systematic review

**DOI:** 10.1177/09697330251333386

**Published:** 2025-04-24

**Authors:** Christina M Lamb, Dimitra V Pouliopoulou, Ken Kirkwood, Kelsey Groenenboom, Megan Kennedy, Edith Pituskin

**Affiliations:** 1349 Athabasca University; University of Toronto; 6221 Western University; 218737 Calgary Foothills Medical Center; 3158 University of Alberta

**Keywords:** Conscience, ethics, moral decision-making, interventions, practice, education

## Abstract

Research indicates that conscience is an asset to healthcare professional’s personal and professional practice. However, little work has been done to support healthcare professionals to use and understand their conscience for moral decision-making. Disparity exists between international and national bodies that value conscience for healthcare professionals and the paucity of practice supports available to formally assist healthcare professionals to openly discuss and then navigate their moral decisions arising from their conscience. Therefore, the purpose of this systematic review was to examine the effectiveness of existing interventions aimed at supporting healthcare professionals to understand and use their conscience for healthcare practice. This review was conducted and reported following the Preferred Reporting Items for Systematic Reviews and Metanalyses. International, interdisciplinary databases including Medline, Embase, PsycINFO, CINAHL, Academic Search Complete, ATLA Religion, Religion and Philosophy Collection, PhilPapers, Scopus and Cochrane Controlled Register of Trials were searched and quantitative as well as qualitative outcomes were reported. We found 11 studies that met the inclusion criteria and underwent data extraction and synthesis. Five interventions were identified that aimed to address aspects of HCP’s conscience. No interventions were identified that aim to support healthcare professionals to understand or use their conscience for moral decision-making in practice. Empirical and humanities research indicates that conscience is essential to healthcare practice, but issues of conscience remain a polarizing experience for many HCPs. Intervention and education-based research are therefore needed to support HCP’s understanding and use of conscience for practice.

## Introduction

Conscience is a unique aspect of being human which allows us to develop our understanding of moral knowledge and subsequently use that knowledge to guide and determine our ethical actions.^1–3^ Specifically, conscience is the innate mechanism one uses to process moral knowledge to act, or to refrain from acting in alliance with morality.

However, in today’s intuitive world, conscience is not a well-known phenomenon, and moral decision-making is relativistic. Consequently, the knowledge one uses to make moral decisions is not consistent from one person to the next. This ambiguity is a challenge for healthcare professionals (HCPs), who need to enact moral decisions daily for and with their patients, in keeping with set clinical codes of ethics and practice standards, and in relation to their own moral well-being and integrity. Yet, these codes and standards are end points, and not representative of the moral and discursive processes necessary to guide HCPs through complex issues of conscience. Issues of conscience can arise for HCPs when their personal or professional articulation of morality conflicts with their professional standards, and the moral views of their colleagues and patients. When this happens, conscientious objections may subsequently follow, and typically manifest as HCPs voicing an objection, based on their conscience, to performing a legal or professional standard of practice that they or their patient may have an ethical conflict with.^4,5^

Yet, ethical conflicts are not necessarily negative. Rather, they can afford opportunities to have fulsome and robust conversations about what course of action ought to be taken.^6^ If viewed pro-actively, conscience can be a positive vehicle for change through which HCPs can be brought together to utilize their collective knowledge to both humanely challenge and simultaneously support one another to find an ethical way forward amidst a moral disagreement. In this way, conscience can be a benefit to HCPs from a relational perspective, because HCPs today are increasingly being met with deeply polarizing and ethically fraught decisions over complex moral questions regarding assisted death; abortion; the use of artificial intelligence; cessation of disproportionate treatment at the end of life; lack of access to palliative care; what the rights of persons as patients and HCPs are and what the role of the HCP is in regard to these matters.^7–13^

Rooting some of the polarizing approaches to conscience from a practical bioethics’ perspective is the notion that conscience is a private, personal or religious phenomenon that has no place in secular, professional contexts.^7,9^ Yet, in the healthcare literature, empirical evidence indicates that conscience is a positive quality that signals HCPs have internal and external, moral sensitivity to ethical issues. Such moral sensitivity is paramount for effective, ethical and person-centred care and for being a morally integrated HCP.^3–5^ And, conscientious decisions are especially relevant in issues of moral gravitas since these decisions often influence an HCP’s moral self as well that of others. Therefore, creating opportunities for HCPs to openly discuss and work through their issues of conscience is an essential for HCPs to navigate their conscience. Although it is up to an individual to inform and then use their conscience, acts of conscience are not always private acts and conscience is not a relativistic phenomenon. Rather, acts of conscience are a process that may move one from an initial or undeveloped moral intuition to the deliberate decision to act or to refrain from acting to stay true to morality.^14^

Yet, acts of conscience can be challenging to articulate and express because they may entail moral disagreement. While it is not within the scope of this paper to investigate the meaning of morality and all the nuances of moral decision-making, it is important to note that moral disagreement is a fixture of today’s healthcare contexts and navigating moral impasses is a necessary skill for HCPs.^6^ Supporting HCPs to understand and use their conscience is one way to help HCPs to navigate their moral disagreements in healthcare practice.

Defining and delineating conscience and the practical manifestation of a conscientious objection aside, issues of conscience can be complex because they are embedded in the extent to which HCPs have acquired moral knowledge and the degree to which they utilize that knowledge to inform their conscience. And perceptions of morality vary according to the meta, normative or practical worldviews one ascribes to. This pluralism does not mean that there is no substantive content to conscience, nor does it indicate that conscience is not relevant to the daily lives of HCPs; rather, it gestures to the fact that there is a substantial, substantive knowledge gap in relation to what conscience is and how it can then be understood and used in healthcare practice.^15^ Further, there is a lack of ethics education in HCP’s formative training, and many HCPs do not know what conscience is, how to inform and develop their conscience and then use it for moral decision-making.^5^ While it is important to attend to what conscience is it is also essential to understand what conscience is not. For instance, conscience and acts of conscience refer to moral matters and should not involve discrimination, such as the refusal of an HCP to care for a patient based on gender or sexual orientation.^11,12^

Rather, conscience is a complex phenomenon. One of the reasons that conscience is not well understood in healthcare contexts is because conscience is an issue for the humanities. Specifically, there is a paucity of evidence in mainstream health and bioethics scholarship in which conscience has been explored from the perspective of an integrated approach to conscience that utilizes the humanities (theology and philosophy), with health science or educational interventions to do so. For instance, although conscience is conceptually central to moral decision-making, theological and philosophical treatment of conscience is far more robust than what exists in the science-based literature.^15^ Yet, little interdisciplinary research and training exists regarding HCPs’ understanding and use of conscience, leaving them poorly equipped to address issues of conscience in healthcare practice.^15–17^

Without addressing conscience from person-centred and interdisciplinary approaches (theology and philosophy) that appreciate conscience as a central and moral aspect of being human, the lack of consideration paid to conscience and its relevance for ethical healthcare practice will remain unchanged for HCPs.

The empirical research that does exist on conscience in healthcare shows that HCPs report conscience as an influential aspect of their ethical practice.^12,13,16^ When able to use their conscience in practice, HCPs have reported decreased stress over practice situations that trouble their conscience; staying true to their personal, professional and patient oriented approaches to care and voicing ethical concerns over patient issues that may otherwise go unnoticed.^18–24^

These findings are consistent with the norms of international organizations which value conscience for the healthcare professions.^22–24^ For instance, conscience and acts of conscience are enshrined as valuable concepts across international medical, nursing and midwifery organizations.^22–24^ At times, conscience is acknowledged in these codes as being core to healthcare provision; in other instances, conscience is formally recognized by way of conscientious objections clauses.^10,11^ Despite this formal recognition across various international and national jurisdictions, little research has been conducted on the meaning and use of conscience for healthcare professional’s moral decision-making.

To start to address the meaning, understanding and use of conscience for HCP’s moral decision-making, we conducted a systematic review study of the effectiveness of interventions on the understanding and use of conscience for medicine, nursing and midwifery across the international, interdisciplinary (bioethics, science, psychology, religious, philosophical and theological), scholarly literature. The research questions that guided this review are:

1. What interventions exist in the scholarly, interdisciplinary literature aimed at enhancing healthcare professionals’ understanding and use of conscience?
2. How effective have the existing interventions been at supporting healthcare professionals’ use and understanding of conscience?^15^

## Methods

### Design

We have previously published a methods paper of the study protocol which was also registered in PROSPERO (CRD42021256943).^15^ As such, we describe the methods in brevity here, and this paper reports the full, systematic reviews study and our findings. Methodologically, this study was guided by the Preferred Reporting Items for Systematic Review; the Meta-analysis and the PRISMA-S checklist to report and document the literature search and the Cochrane Handbook of Systematic Reviews of Interventions (See Supplemental File 1).^15,25–28^

### Search strategy

The search strategy was originally developed and performed by a health sciences librarian following the PRISMA for Searching (PRISMA-S) extension.^26,27^ Utilizing the search strategy we systematically searched the following databases without date restrictions: MEDLINE, Embase, CINAHL, PsycINFO, Scopus, Cochrane Central Register of Controlled Trials (CENTRAL) and PhilPapers. We also conducted a multi-database search in EBSCO including Academic Search Complete, Atla Religion Database, Religion and Philosophy Collection (see Supplemental File 2 for full search strategy).

Articles from the database searches were exported and de-duplicated into the systematic review screening software Covidence.^15,29^ The initial search was conducted in May 2021 with the last search update performed in May, 2024. This manuscript was submitted within a month of conducting the last, updated search.^15^ A manual search of the reference list of the included studies was conducted to identify any additional studies not retrieved in electronic search.

### Eligibility criteria

#### Inclusion criteria

In order to determine which studies were eligible, we adhered to the Population, Intervention, Comparators, Outcomes and Study design (PICOS) framework in the PRISMA (2020) statement.^15,26^ The populations under review consisted of HCPs in clinical practice working across the sub-groups of physicians, nurses and midwives.^15^ Eligible interventions were those that supported HCP’s use and understanding of conscience in some way.^15^ Study designs consisted of interventions in experimental, quasi-experimental and non-experimental studies comprised of clinical trials and qualitative, quantitative or mixed-methods studies.^15^

#### Exclusion criteria

Unpublished articles including theses and dissertations as well as non-English articles were excluded.

### Data collection and extraction

Once the articles were de-duplicated into Covidence, the articles underwent a two-step screening process by a minimum of two, independent researchers.^15^ At least two reviewers independently screened all records by title and abstract and retained articles that met eligibility criteria.^15^ Any discrepancies underwent full review. Included articles then underwent full-text review by two, independent reviewers.^15^ Conflicts were resolved by consensus or the PI.^15^ Full articles that were included were subsequently extracted for data into an Excel spreadsheet corresponding to variables pre-determined by the research team.^15^ The screening and selection process was captured in a PRISMA flowchart.^15^ We extracted data on author’s name, publication year, population characteristics (age, % female and HCP sub-group), country, setting, intervention characteristics, comparator and outcomes of interest.^15^ We did not need to contact any corresponding authors to clarify or request additional information.

For the qualitative studies, the primary outcome of interest was a troubled conscience. A troubled conscience occurs when an HCP is unable to follow their conscience in practice for matters ranging from not being able to follow their core belief or values, time constraints or workload which inhibit them from providing essential patient care.^18^ For the quantitative studies, the primary outcome of interest was stress of conscience, measured by the Stress of Conscience Questionnaire (SCQ). Stress of conscience arises when an HCP is repeatedly unable to address their troubled conscience and can result in burnout, silencing their conscience and a disintegrated sense of one’s moral self.^19,20^ The SCQ assesses 5 domains related to troubled conscience, and each domain is rated from 0 to 25 where higher scores indicate more troubled conscience. The score of each individual domain is added to a create a cumulative score of 0–225. If a study reported multiple follow-up timepoints, we prioritized the ones that were reported as the primary outcome point. If that information was not available, we prioritized the timepoint closer to the end of the intervention.

### Synthesis

#### Data synthesis

For quantitative findings the results of continuous variables were presented as mean (standard deviation, SD). Categorical variables were presented as numbers (percentages). We used descriptive statistics and narrative synthesis to summarize the studies and characteristics of the participants within them. To calculate the effect size, we used the change between the pre and post scores. To calculate the 95% confidence intervals (CIs), we used the standard error (SE). If the standard error was not reported, we calculated it using the standard deviation (SD) and the sample size. All analyses were performed using STATA (Stata Statistical Software: Release 17, StataCorp LLC). We used Digitizelt 2.5.9 to extract data from approximation graphs and figures. For the qualitative synthesis, we captured the findings in text and tables to illustrate the primary concepts of interest and the effects of the interventions.^15,30^

#### Critical appraisal: Assessing risk of bias

Since mixed-methods studies were included in this SR, the data were appraised for methodological quality using the Mixed-Methods Assessment Tool (MMAT) (see Supplemental File 3).^15,31,32^ The MMAT is a critical appraisal tool to ascertain the quality of different study designs included in systematic reviews.^15,32^ Corresponding assessments to the study designs were captured according to 5 criteria for validity and rigour which resulted in a final degree of bias categorized in each study as either low, moderate or high risk of bias.

Each criterion for assessment is rated with either a ‘Yes’, ‘No’, or ‘Can’t tell’ response.^15,32^ For the qualitative and the quantitative studies, if a study had ‘Yes’ across all five categories, then it was deemed as having low risk of bias. If a study had no more than one ‘No’ and no more than one ‘Can’t tell’, it was deemed to have moderate risk of bias. If a study had more than one ‘No’ or ‘Can’t tell’, then it was deemed to have ‘high risk’ of bias. For the mixed-methods studies, we followed the same principle, but if a study had ‘No’ in the last item of the tool that assesses if the study was conducted based on the quality criteria of each component’s design (qualitative and quantitative), it was deemed to have high risk of bias. Given the heterogenous results, we did not perform a meta-analysis or apply the Grading of Recommendations, Assessment, Development and Evaluations approach.^15,33^

## Results

### Search results and studies

**Figure 1.**
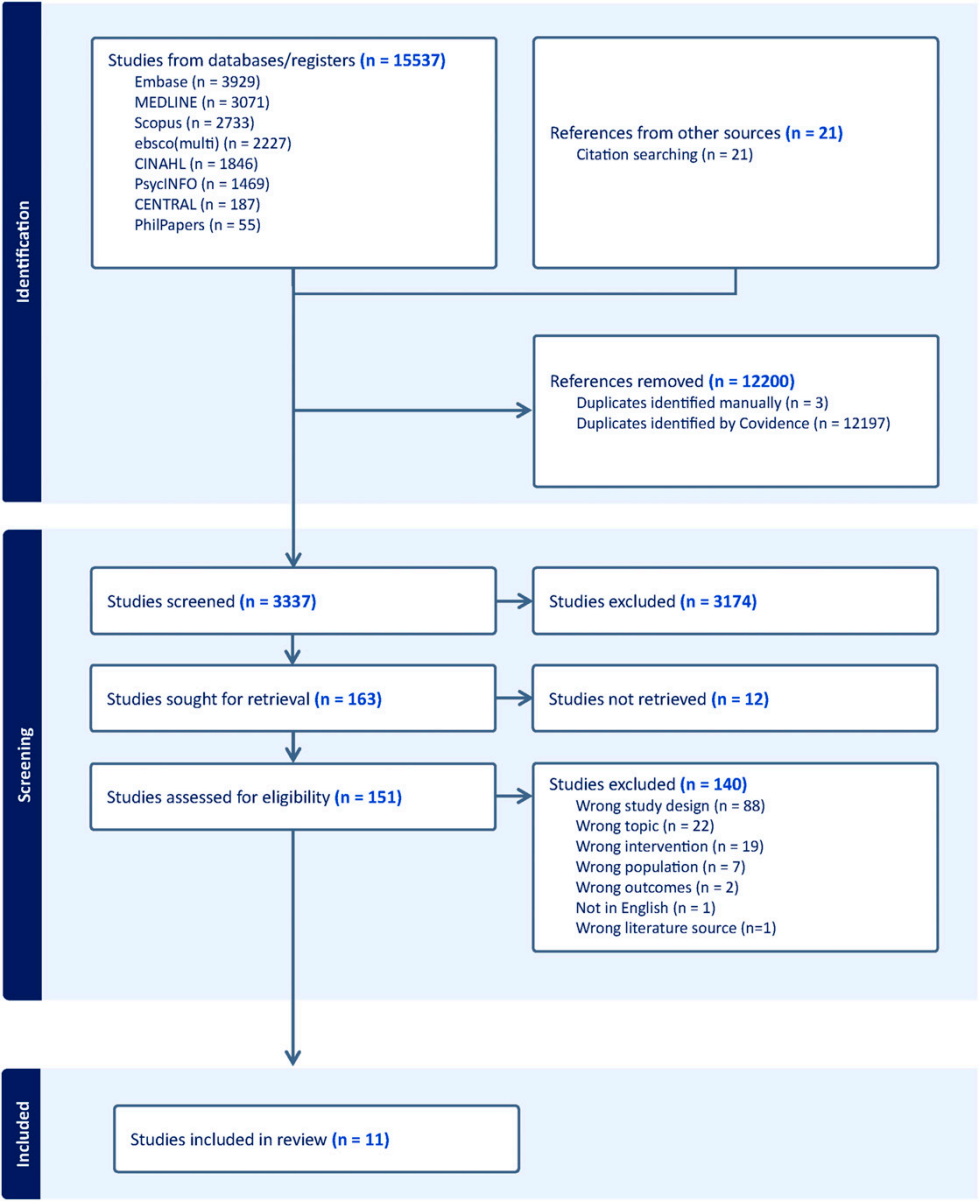
PRISMA flowchart. Source: Page MJ, et al. BMJ 2021;372:n71. doi: 10.1136/bmj.n71.^25^

Across all searches, a total of 15,517 records were identified through database searching; after duplicate records were removed, 3336 records were left for title/abstract screening. Post title and abstract screening, 162 articles were sought for full-text screening of which 11 studies (14 records) were initially eligible for inclusion in the review (see Figure 1 For the PRISMA flowchart). Reasons for exclusion at full-text level are documented in Supplemental File 4. Published between 2013 and 2024, the articles consisted of 10 research studies and 1 clinical trial. Of the 11 studies, 6 were qualitative with 5 belonging to one parent study, and the final 1 was an evaluation of the parent study. The remaining 5 consisted of 1 clinical trial, 1 mixed-methods study and 3 quantitative studies (see Table 1 for study characteristics).^34–44^

**Table 1.** Characteristics of the included studies.

Study	Country	Study design	Population	Sample size	Females (%)	Age	Intervention	Comparator	Outcome	Comments
Ericson-Lidman et al., 2013^34^	Sweden	Participatory action research (PAR)	Nurses in elderly care facility; 22.1 years in healthcare and 8.6 years at the present unit *Mix of RNs, enrolled nurses and nurse assistants	14	N/I	N/I	12 sessions on participant’s troubled conscience related to not responding well to resident’s behaviour (spitting); to brainstorm, describe the problem, identify learning needs, discuss reasonable possible ideas for change and work on action items; two researchers present at every session; duration: 90 min	N/A	Action item effectiveness and healthcare providers’ reflections on observations	Troubled conscience due to troubled patient behaviour
Ericson-Lidman et al., 2015a^35^	Sweden	Participatory action research (PAR)	Nurses in residential care facility; mean level of work experience of 22.7 years in healthcare and 12.2 years at the present unit	13	N/I	N/I	12 sessions on troubled teamwork; brainstormed and described the problem in a deeper way; actions taken to gain increased understanding; listed knowledge and learning needs; discuss reasonable possibilities for change; work out a plan for possible and meaningful actions; actions taken to improve teamwork; actions that were taken were evaluated and revised and new actions were implemented; two researchers as group facilitators, 90 min	N/A	Action item’s effectiveness on teamwork and troubled conscience; a troubled conscience can be a driving force to address improved care for residents	Troubled conscience due to perceptions of deficient teamwork
Ericson-Lidman et al., 2015b^36^	Sweden	Participatory action research (PAR)	Nurses in residential care facility; mean level of work experience of 25.6 years in healthcare and 12.9 years at the present unit	12	N/I		12 sessions on shortcomings on providing sufficient activities for residents; brainstormed and described the problem in a deeper way; actions taken to gain increased understanding and meaningful activities; actions taken and activity actions planned; actions evaluated and revised, and new actions were implemented; two researchers as group facilitators, 90 min	N/A	Action items’ effectiveness on troubled conscience related to activities	Troubled conscience due to providers’ perceptions of not providing sufficient activities for residents
Ericson-Lidman et al., 2015c^37^	Sweden	Participatory action research (PAR)	Nurses consisting of RNs, enrolled nurses, nurse aides and the unit manager in a residential care unit for elderly persons in northern Sweden; mean level of work experience of 22.1 years and 8.6 years at the present unit	13	N/I	N/I	12 interview sessions on scenarios that troubled participants’ conscience; interview findings shared back with participants who prioritized situations that troubled their conscience which arose from perceived shortcomings in providing individualized mealtimes for residents. Problem-based learning (PBL) model was used to generate solutions to address participant’s troubled conscience. Phases consisted of: reflection, taking a stand and intervention. Brainstorming, prioritizing situations, planning for change and activity actions generated. Two researchers as group facilitators, 90 min sessions	N/A	Action items' effectiveness on troubled conscience related to activities	Troubled conscience due to provider’s perceptions of not providing an appropriate meal schedule for residents' needs
Ericson-Lidman et al., 2020^38^	Sweden	Participatory action research (PAR)	Nurses in residential care facility; mean level of work experience of 22.1 years in healthcare and 8.6 years at the present unit	15	N/I	N/I	12 sessions on new time management system that generated troubled conscience for participants. Identifying priority situations that troubled their conscience lead to brainstorming, identifying learning needs and meeting with time management team to discuss possibilities for change. Time management system adapted to participants' needs to alter system to benefit resident’s care needs and ease participant's troubled conscience. Two researchers as group facilitators, 90 min sessions	N/A	Action items’ effectiveness on troubled conscience related to the new time management system	Troubled conscience related to feeling forced to implement a time management system to reduce stress; was perceived as burden that prevents them from providing good care
Juthberg et al., 2016^39^	Sweden	Qualitative - individual interviews	Nurses that participated in PAR interventions from previous studies. Mean level of work experience was 25 years with 11 years in present organization	29	26	N/I	Individual, semi-structured interviews with each participant lasting 10–93 min in length. Interviews recorded, transcribed verbatim, analysed with content analysis resulting in 3 categories and 8 subcategories illustrating participant's experience of the PAR intervention	N/A	PAR intervention’s effectiveness on troubled conscience related to participant's experience with the PAR process	PAR intervention effective and imperfect process to support participants to ease their troubled conscience related to multiple scenarios arising in parent study
Molin et al. (2018)^43^	Sweden	Mixed-methods study	Enrolled nurses (*n* = 12, 24%); registered nurses (*n* = 18, 36%); RN specialized in mental health (n = 6, 12%) other - including unit managers, occupational therapists (OT), physicians and consulting psychiatrists (*n* = 14; 28%)	50	70%	40	Time together (TT): Regular times for TT were established on Mondays through Fridays for a minimum of 5 hours/week total. Patients and staff engaged in joint activities, in either one-on-one or in group sessions. During TT, only one or two staff members dealt with administrative duties on the unit. All other staff were engaged with patients. During TT, the unit was closed to visitors and professionals from outside the unit	N/A	The notes from the participant observations were analysed using qualitative content analysis; Stress of Conscience Questionnaire	The data looked at the percentage of non-overlapping data (PND) statistical technique (Morgan & Morgan 2009). Values lower than 50% indicate an ineffective intervention, 50% to <70% indicate a questionable effect, 70% to <90% indicate an effective intervention and values >90% indicate a very effective intervention
Ericson-Lidman et al., 2017a^42^	Sweden	Participatory action research (PAR)	Registered nurses RNs (*n* = 5, 17%) and nurse assistants NAs (*n* = 24; 83%) participated in the intervention together with their managers	47	90%	52	In individual interviews, the participants were given an opportunity to describe situations that created troubled conscience at work. These situations were summarized and presented during the first PAR session. The participants were then given the opportunity to choose one specific situation among these that generated troubled conscience and proceeded to identify, prioritize and brainstorm about a chosen situation that generated troubled conscience	N/A	Stress of Conscience Questionnaire	Approximation graphs
Johansson et al., 2022^41^	Sweden	feasibility study (randomized controlled)	Nurses, psychologists, psychotherapists, counsellors, occupational therapists and doctors; full- or part-time work with patients and personal experience of work-related stress	32	89%	27–56	Internet-based compassion course (ICOP) based on compassionate mind training (CMT); psychoeducation on the brain with regards to how it regulates emotion, as well as experiential exercises aimed at increasing participant abilities to soothe and care for themselves, developing self-compassion and reducing self-criticism	Internet cognitive–behavioural stress management course (ICB); psychoeducation about stress, behavioural exercises, cognitive restructuring and relaxation techniques	Stress of Conscience Questionnaire	Mean (SD) pre-post per group reported
Edvardsson et al. (2014)^40^	Sweden	Quasi-experimental	Nursing staff, mean work experience of 19 years in healthcare, 19 years in aged care and nine years in the current unit	143	86%	48	The intervention consisted of an interactive step-wise program of knowledge translation, knowledge generation and knowledge dissemination based on the participatory action research cycle	N/A	Stress of Conscience Questionnaire	0–225, higher scores more stress of conscience
Vassbø et al., 2020^44^	Victoria, Australia; Oslo, Norway and Västerbotten, Sweden)	Non-randomized control	61.9% (*N* = 211) were enrolled nurses (ENs), 22.0% (*N* = 75) were registered nurses (RNs), 11.7% (*N* = 40) were care assistants (CAs) and 4.1% (*N* = 14) were allied health staff (AH). The mean work experience in aged care was 13.3 years, while mean work experience in the respective NHs was 10.0 years	341	90%	42	Introductory lecture on the foundations of person-centredness and thriving, followed by nine monthly workshops where staff were encouraged to discuss and reflect on the three dimensions in their care practice. Between the workshops, staff were asked to take part in activities that implemented person-centred care and thriving in clinical practice. The intervention also included an international dissemination seminar where participants from the three countries could exchange experiences and a closing seminar where local participants’ overall experiences were summarized	Offered the same introductory lecture that was given at the intervention NHs. Following this lecture, staff in control NHs continued their practice without further involvement from the research team aside from data collection before and after the intervention	Stress of Conscience Questionnaire	0–225, higher scores more stress of conscience

### Risk of bias assessment

**Figure 2.**
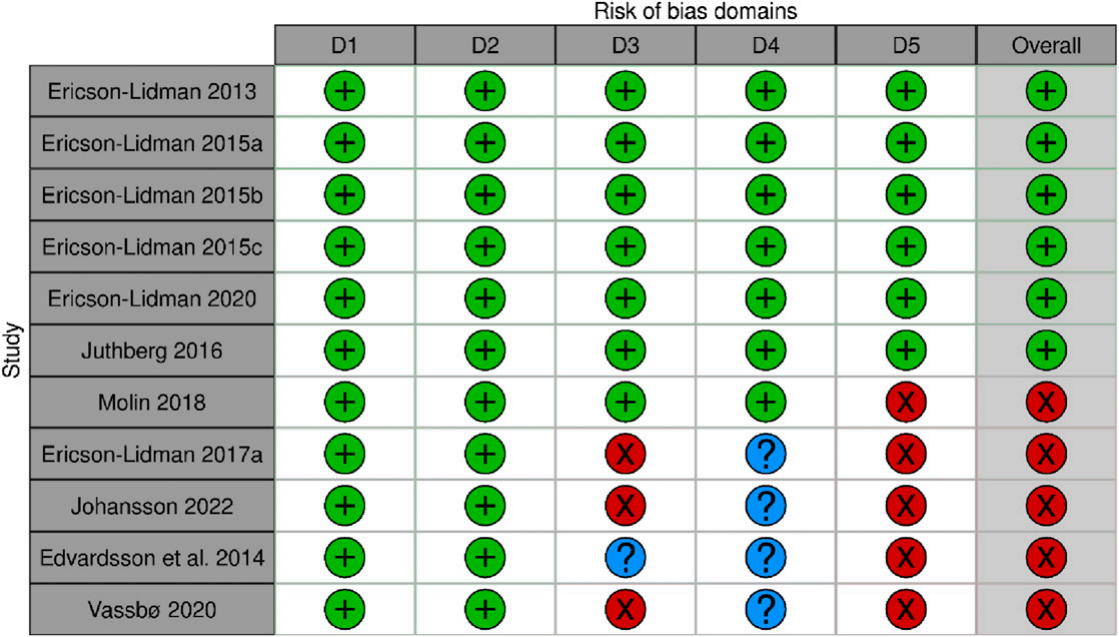
Risk of bias assessment.

Six studies were assessed based on the qualitative criteria, 3 studies were assessed based on the quantitative non-randomized criteria, 1 study was assessed based on the quantitative randomized study criteria and 1 study was assessed based on the mixed-methods criteria. A total of 6 studies (54%) were rated as having low risk of bias; 1(9%) study had moderate, and 4 (36%) studies had high risk of bias. The most common source of bias for the quantitative studies was attrition bias and bias related to the adherence to the intervention. The least common source of bias was selection bias (see Figure 2 for ROB scores).

### Study characteristics

Six qualitative studies were identified that aimed to address healthcare professionals’ troubled conscience across 1 parent study housing 5 of the included qualitative studies.^34–38^ The remaining article was a follow-up study evaluating the effectiveness of the parent study.^39^ The mean sample size in the included studies was 16 (range: 12–29), with mean age listed in 1 study as 51 and the mean percentage of females identified in one study as 26%. All studies were single centred and the participant population comprised of nurses and a unit manager (registered nurses, enrolled nurses, nurse aids and a unit manager).^34–39^

We identified a total of 5 studies that assessed the effectiveness of different types of interventions targeting conscience in a quantitative way. The mean sample size in the included studies was 122 (range: 32–341) and the mean percentage of females was 85%. The average mean age of the HCP was 44 years old (range 40–52). Three studies were single centred, and two studies were multi-centred.^40,42–44^ Two studies included nurses only and 3 studies included a mixed population of inpatient staff including nurses in mental health and registered nurses, unit managers, care assistants, allied health professionals, physicians, psychologists and consulting psychiatrists.^40–44^

### Types of interventions to support HCP’s to use and understand their conscience healthcare practice

We identified 5 different interventions. Ericson-Lindman et al. and Edvardsson et al. used a conscience-targeted participatory action research (PAR) intervention.^34–44^ Ericson-Lindman et al. used individual interviews, where the participants were given an opportunity to describe situations that created troubled conscience at work.^39–44^ These situations were summarized and presented during the first PAR session. The participants were then given subsequent, sessional opportunities to choose a situation that generated their troubled conscience and proceed to identify, prioritize and brainstorm how they would address it.^34–40^

In Edvardsson et al., the intervention consisted of an interactive, step-based program of knowledge translation, knowledge generation and knowledge dissemination based on the PAR cycle.^40^ The PAR process and methodology was used to guide the staff in planning, implementing and evaluating a person-centred practice improvement initiative for their unit.^40^

Vassbo et al. used an intervention based on person-centred care principles in a ‘non-equivalent controlled before–after’ study (Vassbo et al. 2020, p. 1788). Everyone received an introductory lecture to the concepts of person-centredness and thriving.^44^ The control group received no further interventions. The intervention group received nine subsequent monthly workshops where staff discussed and reflected on key aspects of their practice corresponding to core principles in the intervention.^44^ In between attending the workshops, participants were expected to involve themselves in certain activities that utilized person-centred care principles into their healthcare practice.^44^ In Vassbo et al., the intervention also included an international seminar where participants could share their experiences, and a closing seminar enabling local participants to synthesize their experiences.^44^

Molin et al. used ‘time together’, an intervention based on Protected Engagement Time (PET) principles.^43^ Specifically, a set period consisting of 5 protected hours/weeks was set aside for patients and staff to engage in activities together to promote quality interactions between staff and patients that would, over time, reduce staff’s stress of conscience.^43^

Johansson et al. compared two interventions, Compassionate Mind Training and Cognitive Behavioural Therapy (CBT).^41^ Compassionate mind training included psychoeducation on the brain with regards to how it can regulate one’s emotion, experiential exercises aimed at increasing the abilities for one to soothe and care for themselves and learn to develop self-compassion and decrease self-criticism (Johansson et al. 2022, p. 3). Cognitive behavioural therapy entailed psychoeducation regarding stress, the use of behavioural exercises, ‘cognitive restructuring and relaxation techniques’ (Johansson et al., 2022, p. 4).

### Effects of interventions

For the qualitative studies, participants across 4 of the PAR intervention studies relayed some post-interventional improvement in being able to address their troubled conscience.^34,36,38^ In one study, participants reported some lack of empowerment owing to the absence of system-level support.^37^ In the follow-up study, participants shared that while the PAR intervention was helpful, it could be improved upon as it did not address or resolve all identified issues that troubled their consciences.^39^

**Figure 3.**
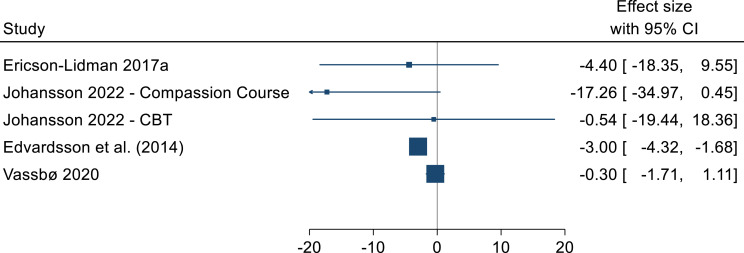
Stress of conscience (pre-post) effect sizes.

For the quantitative studies, four studies reported the effectiveness of the interventions using the pre and post scores on SCQ.^40–42,44^ The pre and post effect sizes for the intervention arms are summarized in Figure 3. All studies reported a reduction in the SCQ score post intervention, ranging from 0.3 to 17.26 points. One study reported a statistically significant improvement (ES: −3, 95% CI: −4.32 to −1.68).^40^ One study reported the results as the percentage of non-overlapping data (pre-post) in three different inpatient centres.^43^ The percentage of the overlapping data indicated that in none of the 3 centres the intervention was successful. The majority of the included studies had very high attrition rates and very low adherence rates.

### Differences in sub-groups: Medicine, nursing and midwifery

There were no studies that examined midwives and the majority of the included studies explored the experiences of nurses and so we were not able to compare the effects of the interventions across the sub-groups.

## Discussion

### Overview of the results

Findings from Ericson-Lindman et al. and Strandberg illustrate that although nurse HCPs experienced a troubled conscience related to various patient care issues in practice encompassing time constraints, challenging patient behaviour and HCPs lack of teamwork and providing activities to residents, these HCPs lacked concrete processes empowering them to articulate the ethical issues undergirding them to then formally address them.^34–39^ Following on from these findings, researchers recommended institutional-level changes to effect potential, positive change in this area.^37^ All the quantitative studies reported a reduction in the SCQ score post intervention, ranging from 0.3 to 17.26 points, but only one study reported a statistically significant improvement. However, all the quantitative studies had serious methodological shortcomings.

The quantitative studies bore resemblance to the qualitative work in terms of target population. Almost all the studies included in this review addressed nurses with exception for two studies in which physicians were included.^41,42^ The interventions consisted of conceptual, educational and discursive interventions. While all the interventions related to conscience in some way, no interventions were explicitly directed at supporting HCPs to understand and use their conscience. The studies included in this review illustrate that little work and few effective strategies exist at the relational, collegial, professional and systems levels to assist HCPs to effectively understand and subsequently use their conscience in care practice.

### Relevant research

For over a decade, insightful research has consistently been emerging from Scandinavia related to HCPs issues of conscience, much of which focuses on nurses. Ten years ago, little empirical research existed on the concept of conscience and conscientious objection in healthcare.^5^ Today, copious amounts of descriptive research worldwide have emerged, focussing mostly on the prevalence of stress of conscience and conscientious objections and the use of measurement tools to measure stress of conscience.^45–47^ Yet, our review identified very few interventions that promote and support HCPs to understand and use their conscience for healthcare practice, and, none of the included interventions targeted HCPs’ formative or on-going education to address HCPs’ conscience issues. The lack of international and interdisciplinary support available in the literature is concerning given that conscience is central to ethical healthcare delivery, but very little has been done to pro-actively include interventions on conscience in the healthcare or bioethics literature.^4,5^ The fact that the interventions we identified do not address the issues of moral gravitas that HCPs are contending with is a problem because it may mean that HCP’s moral concerns are being overlooked. Given that conscience has historically been taken up in moral philosophy and theological ethics, it is time that the health sciences start engaging with the humanities to more fulsomely articulate what conscience is, and then how to use it for moral decisions making in healthcare contexts.

Interdisciplinary, interventional research is therefore needed to investigate how HCPs can be supported over issues of conscience, and especially issues of moral gravitas given that our review did not locate any studies using interventions to support HCPs through articulating what their issues of moral gravitas are, and how they could be supported to address them. For instance, moral philosophers and theologians are well versed in moral disagreement and HCPs may benefit from having interdisciplinary discussions or formal training with them to both articulate and process through what their issues of conscience are to then address them.^6^

Relevantly, education research in a European nursing context has been conducted on the implications that a lack of moral development and training has on nursing practice.^48–51^ Recent studies with nursing students on their experience of formative, ethics education show respondents are frustrated with the lack of ethics education they receive in training, which they report leave them ill-equipped to address the ethical in clinical practice.^48^ Yet, within and across this scholarship, no work has been done to explore how HCPs understand and use their conscience from an epistemological lens.^15^ Without an epistemological basis for understanding what conscience is and the moral knowledge requisite to use conscience, HCPs will not be equipped to address the ethical issues arising in their clinical, ethical practice. And this is an ethical problem because moral decision-making is clinically driven.

### Clinical implications for ethical practice

Moral decision-making is a central component of professional healthcare practice. Specifically, medicine, nursing and midwifery are internationally recognized as well established, ethical professions where conscience has a fundamental role to play.^22–24^ At the same time, increasing numbers of research-based evidence indicate that conscience is essential to HCPs welfare, to patient-centred, caring, person-centred practice and the retention of HCPs.^10,12,23^ Importantly, this study highlights the lack of formal interventions in place for supporting HCPs to understand and use their conscience for moral decision-making in healthcare practice. Moreover, little work has been done to integrate, mitigate and address HCP’s issues of conscience when their conscience prompts them to act, even when their professional regulatory bodies make provisions for them to do so.^5^ As such, our findings indicate that education -based research is a necessary next step to ascertain whether HCPs across disciplines and international contexts are receiving formative and on-going ethics education support to articulate and address their issues of conscience in preparation for clinical practice.

### Limitations

The robustness of our results is dependent on the quality of the included studies. Most of the studies did not find a significant effect of the conscience-targeted interventions in improving stress of conscience. However, none of the included interventions were explicitly directed at supporting HCPs to understand and use their conscience, hence the lack of effectiveness could be attributed to the lack of structured and relevant intervention based on utilizing conscience as an antecedent to moral decision-making. Moreover, it is possible that these results reflect an overall lack of methodological rigour and feasibility of the interventions rather than a lack of effect. Specifically, only two of the studies included in the quantitative analysis had a control group; only one of which was randomized where an active intervention was used instead of a control group. Another limitation was the small sample sizes. While small sample sizes are appropriate in qualitative research, the results of the quantitative studies are imprecise and do not allow for definitive conclusions to be made.^52^

Further limitations in the included studies were the lack of adherence and the high attrition rates. When designing an intervention, its usability, applicability and fidelity in the targeted clinical setting need consideration. The reported attrition and adherence rate possibly indicate that more planning and time needed to be spent in pre-test phases before testing the intervention’s effectiveness.

Another limitation is the lack of a minimally clinically important difference (MCID) for the SCQ tool. It is currently unclear what effect size would be perceived by HCPs as a clinically important improvement in troubled conscience when measured by the SCQ. Although our qualitative synthesis offers an insight into HCP’s perceptions, when designing quantitative interventions, it is important to have standardized, validated measures and cut-off scores to measure the statistical significance as well as the clinical significance of the results. A small improvement in a clinical score might be statistically significant but irrelevant if it is not perceived as significant by the target population. Therefore, future studies should focus on establishing an MCID, and incorporating it in the study design and reporting, to better assess the effects of conscience-targeted interventions in HCPs.

## Conclusion

In summary, this study identifies that few interventions exist aimed at directly supporting HCPs to understand what conscience is or to use it for moral decision-making. At the same time, issues of conscience remain a challenging phenomenon for HCPs and empirical research worldwide indicates that conscience is essential to healthcare practice. Research is therefore needed to lead this complex area of bioethics forward to start to address the ethical challenges arising from HCP’s issues of conscience in healthcare contexts.

## Supplemental Material

Supplemental Material - Effectiveness of interventions on conscience: Findings of a systematic review Supplemental Material for Effectiveness of interventions on conscience: Findings of a systematic review by Christina M Lamb, Dimitra V Pouliopoulou, Ken Kirkwood, Kelsey Groenenboom, Megan Kennedy and Edith Pituskin in Nursing Ethics.

## Data Availability

Data sharing not applicable to this article as no datasets were generated or analysed during the current study.
