# Supplementary material for: Effectiveness of interventions on conscience: Findings of a systematic review
Source: Nurs Ethics. 2025 Apr 24;32(7):2323–46. doi: 10.1177/09697330251333386 (PMC12550205; doi:10.1177/09697330251333386)
Supplement: Supplemental Material - Effectiveness of interventions on conscience: Findings of a systematic review [file sj-pdf-1-nej-10.1177_09697330251333386.pdf]

Supplementary File

Title: Effectiveness of interventions on healthcare professionals' understanding and use of conscience: A systematic review.

Table of Contents

Supplementary File 1. Prisma Checklist 2
Supplementary File 2. Search Strategy 4
Supplementary File 3. MMAT Tool 12
Supplementary File 4. Reasons for exclusion 13
References 17

Supplementary File 1. Prisma Checklist

Section and Topic	Item #	Checklist item	Location where item is reported
**TITLE**			
Title	1	Identify the report as a systematic review.	Page 1
**ABSTRACT**			
Abstract	2	See the PRISMA 2020 for Abstracts checklist.	Page 1
**INTRODUCTION**			
Rationale	3	Describe the rationale for the review in the context of existing knowledge.	Page 2-4
Objectives	4	Provide an explicit statement of the objective(s) or question(s) the review addresses.	Page 4
**METHODS**			
Eligibility criteria	5	Specify the inclusion and exclusion criteria for the review and how studies were grouped for the syntheses.	Page 4-5
Information sources	6	Specify all databases, registers, websites, organisations, reference lists and other sources searched or consulted to identify studies. Specify the date when each source was last searched or consulted.	Page 5
Search strategy	7	Present the full search strategies for all databases, registers and websites, including any filters and limits used.	Supplement
Selection process	8	Specify the methods used to decide whether a study met the inclusion criteria of the review, including how many reviewers screened each record and each report retrieved, whether they worked independently, and if applicable, details of automation tools used in the process.	Page 4-6
Data collection process	9	Specify the methods used to collect data from reports, including how many reviewers collected data from each report, whether they worked independently, any processes for obtaining or confirming data from study investigators, and if applicable, details of automation tools used in the process.	Page 6
Data items	10a	List and define all outcomes for which data were sought. Specify whether all results that were compatible with each outcome domain in each study were sought (e.g. for all measures, time points, analyses), and if not, the methods used to decide which results to collect.	Page 6
	10b	List and define all other variables for which data were sought (e.g. participant and intervention characteristics, funding sources). Describe any assumptions made about any missing or unclear information.	Page 6
Study risk of bias assessment	11	Specify the methods used to assess risk of bias in the included studies, including details of the tool(s) used, how many reviewers assessed each study and whether they worked independently, and if applicable, details of automation tools used in the process.	Page 7-8
Effect measures	12	Specify for each outcome the effect measure(s) (e.g. risk ratio, mean difference) used in the synthesis or presentation of results.	Page 7
Synthesis methods	13a	Describe the processes used to decide which studies were eligible for each synthesis (e.g. tabulating the study intervention characteristics and comparing against the planned groups for each synthesis (item #5)).	Page 7
	13b	Describe any methods required to prepare the data for presentation or synthesis, such as handling of missing summary statistics, or data conversions.	Page 7
	13c	Describe any methods used to tabulate or visually display results of individual studies and syntheses.	Page 7
	13d	Describe any methods used to synthesize results and provide a rationale for the choice(s). If meta-analysis was performed, describe the model(s), method(s) to identify the presence and extent of statistical heterogeneity, and software package(s) used.	Page 7
	13e	Describe any methods used to explore possible causes of heterogeneity among study results (e.g. subgroup analysis, meta-regression).	N/A
	13f	Describe any sensitivity analyses conducted to assess robustness of the synthesized results.	N/A
Reporting bias	14	Describe any methods used to assess risk of bias due to missing results in a synthesis	Page 7-8

Section and Topic	Item #	Checklist item	Location where item is reported
assessment		(arising from reporting biases).	
Certainty assessment	15	Describe any methods used to assess certainty (or confidence) in the body of evidence for an outcome.	N/A
**RESULTS**			
Study selection	16a	Describe the results of the search and selection process, from the number of records identified in the search to the number of studies included in the review, ideally using a flow diagram.	Page 8
	16b	Cite studies that might appear to meet the inclusion criteria, but which were excluded, and explain why they were excluded.	N/A
Study characteristics	17	Cite each included study and present its characteristics.	Page 9-12
Risk of bias in studies	18	Present assessments of risk of bias for each included study.	Page 9, Figure 2
Results of individual studies	19	For all outcomes, present, for each study: (a) summary statistics for each group (where appropriate) and (b) an effect estimate and its precision (e.g. confidence/credible interval), ideally using structured tables or plots.	Page 11-12
Results of syntheses	20a	For each synthesis, briefly summarise the characteristics and risk of bias among contributing studies.	N/A
	20b	Present results of all statistical syntheses conducted. If meta-analysis was done, present for each the summary estimate and its precision (e.g. confidence/credible interval) and measures of statistical heterogeneity. If comparing groups, describe the direction of the effect.	N/A
	20c	Present results of all investigations of possible causes of heterogeneity among study results.	N/A
	20d	Present results of all sensitivity analyses conducted to assess the robustness of the synthesized results.	N/A
Reporting biases	21	Present assessments of risk of bias due to missing results (arising from reporting biases) for each synthesis assessed.	N/A
Certainty of evidence	22	Present assessments of certainty (or confidence) in the body of evidence for each outcome assessed.	N/A
**DISCUSSION**			
Discussion	23a	Provide a general interpretation of the results in the context of other evidence.	Page 12-13
	23b	Discuss any limitations of the evidence included in the review.	Page 14-15
	23c	Discuss any limitations of the review processes used.	Page 14-15
	23d	Discuss implications of the results for practice, policy, and future research.	Page 13-15
**OTHER INFORMATION**			
Registration and protocol	24a	Provide registration information for the review, including register name and registration number, or state that the review was not registered.	Page 4
	24b	Indicate where the review protocol can be accessed, or state that a protocol was not prepared.	Page 4
	24c	Describe and explain any amendments to information provided at registration or in the protocol.	Page 8
Support	25	Describe sources of financial or non-financial support for the review, and the role of the funders or sponsors in the review.	Title page
Competing interests	26	Declare any competing interests of review authors.	Title page
Availability of data, code and other materials	27	Report which of the following are publicly available and where they can be found: template data collection forms; data extracted from included studies; data used for all analyses; analytic code; any other materials used in the review.	Title page

From: Page MJ, McKenzie JE, Bossuyt PM, Boutron I, Hoffmann TC, Mulrow CD, et al. The PRISMA 2020 statement: an updated guideline for reporting systematic reviews. BMJ 2021;372:n71. doi: 10.1136/bmj.n71

Supplementary File 2. Search Strategy

Full Search Strategy

Ovid MEDLINE(R) ALL <1946 to August 28, 2023>

1 conscience/ 1689
2 (conscience? or "conscientious* object*").mp. 4086
3 empirical ethics.mp. 191
4 1 or 2 or 3 4276
5 health personnel/ or exp nurses/ or exp nurse practitioners/ or exp nurse specialists/ or exp nurses, pediatric/ or exp physicians/ 331055
6 (doctor* or physician* or "general practitioner*" or GP or surgeon* or anesthesiologist* or cardiologist* or dermatologist* or radiologist* or internist* or geneticist* or neurologist* or obstetrician* or gyn?ecologist* OBGYN or OB-GYN or ophthalmologist* or pathologist* or p?ediatrician* or psychiatrist* or oncologist* or urologist* or allergist* or endocrinologist* or gastroenterologist* or geriatrician* or hospitalist* or nephrologist* or pulmonologist* or rheumatologist* or clinician* or nurse* or midwife* or midwives or ((healthcare or "health care" or health or medical or nursing) adj3 (professional* or personnel or practitioner* or provider*))).mp. 2185826
7 5 or 6 2188375
8 (intervention* or train* or educat* or workshop* or seminar* or curricul* or coach* or mentor* or experiment* or tool* or interview* or "focus group" or review* or screening or questionnaire* or survey* or perception* or implication* or barrier* or facilitat* or obstacle* or encourag* or impediment* or impede* or challeng* or obstruct* or hurdle or experience* or perceive* or perspective* or perception* or "self-report" or trial* or test* or "quality improvement*" or "quality assurance" or "patient safety").mp. 17067869
9 ((QI or "quality improvement*") adj6 (intervention* or initiative* or strateg* or program* or campaign* or implement*)).mp. 22962
10 ((Practice or behavio* or organizational) adj3 chang*).mp. 106849
11 education/ or curriculum/ or education, distance/ or exp education, professional/ or exp education, continuing/ or exp education, graduate/ or exp education, medical/ or exp education, nursing/ or exp inservice training/ or mentoring/ 398995
12 focus groups/ or interviews as topic/ or "surveys and questionnaires"/ or health care surveys/ or self report/ 702990
13 Quality Improvement/ 32722
14 (((research or knowledge or evidence) adj2 (uptake or "use" or diffus$ or disseminat$ or utiliz$ or utilis$ or transfer$ or translat$ or implement$ or adopt$)) or (innovation$ adj2 adopt$) or (innovation$ adj2 disseminat$) or "research into practice" or "evidence into practice" or "knowledge to action" or "know do gap" or (knowledge adj (mobilization or exchange)) or "translational science" or "implementation science").ti,ab. 89234
15 exp "Diffusion of Innovation"/ or Organizational Innovation/ 45386
16 (evidence-informed adj (healthcare or health care or decision making)).ti,ab. 307

17 (guideline* adj2 (introduc*or issu* or impact or effect* or distribut* or adher* or compl* or utiliz* or utilis* or "use" or uptake or diffuse* or transfer* or implement* or translat* or disseminat* or adopt*)).ti,ab. 25010

18 exp *Evidence-Based Practice/ or Information dissemination/ or Knowledge management/ or Health Knowledge, Attitudes, Practice/ 179288

19 or/8-18 17147624

20 4 and 7 and 19 1039

21 limit 20 to english language 900

22 (editorial or note or letter or opinion).pt. 1888566

23 21 not 22 885

Embase <1974 to 2023 August 29>

1 conscience/ 2769

2 (conscience? or "conscientious* object*").mp. 5035

3 empirical ethics.mp. 224

4 1 or 2 or 3 5258

5 health care personnel/ or clinician/ 255734

6 exp nurse/ or exp advanced practice nurse/ or exp nurse practitioner/ or exp nurse specialist/ 217483

7 exp physician/ or exp cardiologist/ or exp dermatologist/ or exp gynecologist/ or exp hematologist/ or exp obstetrician/ or exp oncologist/ or exp orthopedic specialist/ or exp pathologist/ or exp pediatrician/ or exp radiologist/ or exp surgeon/ or exp urologist/ or exp venereologist/ 966676

8 exp midwife/ 35817

9 (doctor* or physician* or "general practitioner*" or GP or surgeon* or anesthesiologist* or cardiologist* or dermatologist* or radiologist* or internist* or geneticist* or neurologist* or obstetrician* or gyn?ecologist* OBGYN or OB-GYN or ophthalmologist* or pathologist* or p?ediatrician* or psychiatrist* or oncologist* or urologist* or allergist* or endocrinologist* or gastroenterologist* or geriatrician* or hospitalist* or nephrologist* or pulmonologist* or rheumatologist* or clinician* or nurse* or midwife* or midwives or ((healthcare or "health care" or health or medical or nursing) adj3 (professional* or personnel or practitioner* or provider*))).mp. 3223752

10 5 or 6 or 7 or 8 or 9 3251918

11 (intervention* or train* or educat* or workshop* or seminar* or curricul* or coach* or mentor* or experiment* or tool* or interview* or "focus group" or review* or screening or questionnaire* or survey* or perception* or implication* or barrier* or facilitat* or obstacle* or encourag* or impediment* or impede* or challeng* or obstruct* or hurdle or experience* or perceive* or perspective* or perception* or "self-report" or trial* or test* or "quality improvement*" or "quality assurance" or "patient safety").mp. 23193493

12 ((QI or "quality improvement*") adj6 (intervention* or initiative* or strateg* or program* or campaign* or implement*)).mp. 35434

13 ((Practice or behavio* or organizational) adj3 chang*).mp. 158248

14 education/ or continuing education/ or curriculum/ or doctoral education/ or health education/ or in service training/ or lifelong learning/ or masters education/ or exp medical education/ or mentoring/ or postdoctoral education/ or postgraduate education/ or refresher course/ or vocational education/ 962724

15 exp clinical education/ 17133

16 exp nursing education/ 89407

17 questionnaire/ or open ended questionnaire/ or structured questionnaire/ 925635

18 health survey/ 230104

19 self report/ 154390

20 total quality management/ 90971

21 (((research or knowledge or evidence) adj2 (uptake or "use" or diffus$ or disseminat$ or utiliz$ or utilis$ or transfer$ or translat$ or implement$ or adopt$)) or (innovation$ adj2 adopt$) or (innovation$ adj2 disseminat$) or "research into practice" or "evidence into practice" or "knowledge to action" or "know do gap" or (knowledge adj (mobilization or exchange)) or "translational science" or "implementation science").ti,ab. 114935

22 mass communication/ 15236

23 (evidence-informed adj (healthcare or health care or decision making)).ti,ab. 339

24 (guideline* adj2 (introduc*or issu* or impact or effect* or distribut* or adher* or compl* or utiliz* or utilis* or "use" or uptake or diffuse* or transfer* or implement* or translat* or disseminat* or adopt*)).ti,ab. 39460

25 organization* innovat*.ti,ab. 256

26 evidence based practice/ or exp evidence based medicine/ or evidence based nursing/ 1803005

27 information dissemination/ 25023

28 knowledge management/ 2168

29 attitude to health/ 130877

30 or/11-29 23358340

31 4 and 10 and 30 1341

32 limit 31 to (books or chapter or conference abstract or conference paper or "conference review" or editorial or letter or note) 241

33 31 not 32 1100

34 limit 33 to english language 945

APA PsycInfo <1806 to August Week 3 2023>

1 conscience/ 973

2 (conscience? or "conscientious* object*").mp. 4569

3 empirical ethics.mp. 130

4 1 or 2 or 3 4699

5 health personnel/ or exp medical personnel/ 114694

6 exp nurses/ or exp physicians/ 84689

7 clinicians/ 13534

8 midwifery/ 1776

9 (doctor* or physician* or "general practitioner*" or GP or surgeon* or anesthesiologist* or cardiologist* or dermatologist* or radiologist* or internist* or geneticist* or neurologist* or obstetrician* or gyn?ecologist* OBGYN or OB-GYN or ophthalmologist* or pathologist* or p?ediatrician* or psychiatrist* or oncologist* or urologist* or allergist* or endocrinologist* or gastroenterologist* or geriatrician* or hospitalist* or nephrologist* or pulmonologist* or rheumatologist* or clinician* or nurse* or midwife* or midwives or ((healthcare or "health care" or health or medical

or nursing) adj3 (professional* or personnel or practitioner* or provider*))).mp. 460884
10 5 or 6 or 7 or 8 or 9 463617
11 (intervention* or train* or educat* or workshop* or seminar* or curricul* or coach* or mentor* or experiment* or tool* or interview* or "focus group" or review* or screening or questionnaire* or survey* or perception* or implication* or barrier* or facilitat* or obstacle* or encourag* or impediment* or impede* or challeng* or obstruct* or hurdle or experience* or perceive* or perspective* or perception* or "self-report" or trial* or test* or "quality improvement*" or "quality assurance" or "patient safety").mp. 4213922
12 ((QI or "quality improvement*") adj6 (intervention* or initiative* or strateg* or program* or campaign* or implement*)).mp. 2432
13 ((Practice or behavio* or organizational) adj3 chang*).mp. 81206
14 education/ or curriculum/ or distance education/ or exp personnel training/ 97458
15 exp medical education/ or nursing education/ 34229
16 higher education/ or graduate education/ or postgraduate training/ or undergraduate education/ 32207
17 mentor/ or professional development/ 31045
18 exp qualitative methods/ or exp focus group/ 21212
19 interviews/ 12818
20 exp surveys/ or exp questionnaires/ 41187
21 self-report/ 22894
22 (((research or knowledge or evidence) adj2 (uptake or "use" or diffus$ or disseminat$ or utiliz$ or utilis$ or transfer$ or translat$ or implement$ or adopt$)) or (innovation$ adj2 adopt$) or (innovation$ adj2 disseminat$) or "research into practice" or "evidence into practice" or "knowledge to action" or "know do gap" or (knowledge adj (mobilization or exchange)) or "translational science" or "implementation science").ti,ab. 44392
23 innovation/ 17914
24 (evidence-informed adj (healthcare or health care or decision making)).ti,ab. 69
25 (guideline* adj2 (introduc*or issu* or impact or effect* or distribut* or adher* or compl* or utiliz* or utilis* or "use" or uptake or diffuse* or transfer* or implement* or translat* or disseminat* or adopt*)).ti,ab. 4104
26 evidence based practice/ 20978
27 information dissemination/ or knowledge transfer/ 5749
28 knowledge management/ 4746
29 health knowledge/ or health attitudes/ 19501
30 or/11-29 4237149
31 4 and 10 and 30 517
32 limit 31 to (chapter or "column/opinion" or "comment/reply" or editorial or letter) 69
33 31 not 32 448
34 limit 33 to english language 374

CINAHL via EBSCOhost (1936 - Aug 30, 2023)

S1 (MH "Conscience") OR (conscience# or "conscientious* object*" or "empirical ethics") 2,157
S2 ((MH "Health Personnel") OR (MH "Midwives+") OR (MH "Nurses+") OR (MH "Nurses by Educational Level+") OR (MH "Nurses by Role+") OR (MH "Advanced Practice Nurses+") OR (MH "Nurse Consultants+") OR (MH "Nurses by Specialty+") OR (MH "Pediatric Nurse Practitioners+") OR (MH "Nurses, Other+") OR (MH "Physicians+") OR (MH "Pathologists+")) OR ((doctor* or physician* or "general practitioner*" or GP or surgeon* or anesthesiologist* or cardiologist* or dermatologist* or radiologist* or internist* or geneticist* or neurologist* or obstetrician* or gyn#ecologist* or OBGYN or OB-GYN or ophthalmologist* or pathologist* or p#ediatrician* or psychiatrist* or oncologist* or urologist* or allergist* or endocrinologist* or gastroenterologist* or geriatrician* or hospitalist* or nephrologist* or pulmonologist* or rheumatologist* or clinician* or nurse* or midwife* or midwives) OR ((healthcare or "health care" or health or medical or nursing) N3 (professional* or personnel or practitioner* or provider*))) 1,382,156
S3 (intervention* or train* or educat* or workshop* or seminar* or curricul* or coach* or mentor* or experiment* or tool* or interview* or "focus group" or review* or screening or questionnaire* or survey* or perception* or implication* or barrier* or facilitat* or obstacle* or encourag* or impediment* or impede* or challeng* or obstruct* or hurdle or experience* or perceive* or perspective* or perception* or "self-report" or trial* or test* or "quality improvement*" or "quality assurance" or "patient safety") OR ((QI or "quality improvement*") N6 (intervention* or initiative* or strateg* or program* or campaign* or implement*)) OR ((Practice or behavio* or organizational) N3 chang*) OR ((MH "Education") OR (MH "Curriculum+") OR (MH "Education, Clinical") OR (MH "Education, Health Sciences+") OR (MH "Education, Baccalaureate+") OR (MH "Education, Associate+") OR (MH "Education, Continuing+") OR (MH "Education, Graduate+") OR (MH "Education, Doctoral+") OR (MH "Education, Masters+") OR (MH "Education, Nursing, Graduate+") OR (MH "Education, Post-Doctoral+") OR (MH "Education, Medical+") OR (MH "Education, Midwifery") OR (MH "Education, Nursing+") OR (MH "Education, Nursing, Baccalaureate+") OR (MH "Internship and Residency")) OR (MH "Mentorship") OR ((MH "Focus Groups") OR (MH "Interviews+") OR (MH "Self Report") OR (MH "Surveys+")) OR (MH "Questionnaires+") OR (MH "Quality Improvement+") OR (TI ((((research or knowledge or evidence) N2 (uptake or "use" or diffus* or disseminat* or utiliz* or utilis* or transfer* or translat* or implement* or adopt*)) or (innovation* N2 adopt*) or (innovation* N2 disseminat*) or "research into practice" or "evidence into practice" or "knowledge to action" or "know do gap" or (knowledge N1 (mobilization or exchange)) or "translational science" or "implementation science")) OR AB ((((research or knowledge or evidence) N2 (uptake or "use" or diffus* or disseminat* or utiliz* or utilis* or transfer* or translat* or implement* or adopt*)) or (innovation* N2 adopt*) or (innovation* N2 disseminat*) or "research into practice" or "evidence into practice" or "knowledge to action" or "know do gap" or (knowledge N1 (mobilization or exchange)) or "translational science" or "implementation science"))) OR (MH "Diffusion of Innovation+") OR (evidence-informed N1 (healthcare or health care or decision making)) OR (TI (guideline* N2 (introduc* or issu* or impact or effect* or distribut* or adher* or compl* or utiliz* or utilis* or "use" or uptake or diffuse* or transfer* or implement* or translat* or disseminat* or adopt*)) OR AB (guideline* N2 (introduc*or issu* or impact or effect* or distribut* or adher* or compl* or utiliz* or utilis* or "use" or uptake or diffuse* or transfer* or implement* or translat* or disseminat* or adopt*))) Expanders - Apply equivalent subjects 4,489,478

S4 (MH "Nursing Practice, Evidence-Based") OR ((MH "Professional Practice, Evidence-Based") OR (MH "Medical Practice, Evidence-Based")) OR (TI "evidence based practice" OR AB "evidence based practice") OR (MH "Knowledge Management") OR ((MH "Attitude of Health Personnel") OR (MH "Midwife Attitudes") OR (MH "Physician Attitudes") OR (MH "Nurse Attitudes")) OR ((MH "Health Knowledge") OR (MH "Professional Knowledge+")) 246,149
S5 S3 OR S4 4,534,547
S6 S1 AND S2 AND S5 707
S7 S1 AND S2 AND S5 Limiters - Scholarly (Peer Reviewed) Journals; English Language
594

Academic Search Complete, Atla Religion Database, Religion and Philosophy Collection (Multi-Database Search) via EBSCOhost (keyword only)
S1 conscience* or "conscientious* object*" or "empirical ethics" Expanders - Apply equivalent subjects 25,876
S2 (doctor* or physician* or "general practitioner*" or GP or surgeon* or anesthesiologist* or cardiologist* or dermatologist* or radiologist* or internist* or geneticist* or neurologist* or obstetrician* or gyn#ecologist* or OBGYN or OB-GYN or ophthalmologist* or pathologist* or p#ediatrician* or psychiatrist* or oncologist* or urologist* or allergist* or endocrinologist* or gastroenterologist* or geriatrician* or hospitalist* or nephrologist* or pulmonologist* or rheumatologist* or clinician* or nurse* or midwife* or midwives) OR ((healthcare or "health care" or health or medical or nursing) N3 (professional* or personnel or practitioner* or provider*))
1,880,659
S3 intervention* or train* or educat* or workshop* or seminar* or curricul* or coach* or mentor* or experiment* or tool* or interview* or "focus group" or review* or screening or questionnaire* or survey* or perception* or implication* or barrier* or facilitat* or obstacle* or encourag* or impediment* or impede* or challeng* or obstruct* or hurdle or experience* or perceive* or perspective* or perception* or "self-report" or trial* or test* or "quality improvement*" or "quality assurance" or "patient safety" Expanders - Apply equivalent subjects 21,048,394
S4 (guideline* N2 (introduc* or issu* or impact or effect* or distribut* or adher* or compl* or utiliz* or utilis* or "use" or uptake or diffuse* or transfer* or implement* or translat* or disseminat* or adopt*)) OR "evidence based practice" Expanders - Apply equivalent subjects 75,483
S5 S1 AND S2 AND (S3 OR S4) 1,133
S6 S1 AND S2 AND (S3 OR S4) Limiters - Peer Reviewed, English
965

Scopus
((TITLE-ABS-KEY (conscience* OR "conscientious* object*" OR "empirical ethics")) AND (TITLE-ABS-KEY ((doctor* OR physician* OR "general practitioner*" OR gp OR surgeon* OR anesthesiologist* OR cardiologist* OR dermatologist* OR radiologist* OR internist* OR geneticist* OR neurologist* OR obstetrician* OR gyn?ecologist* AND obgyn OR ob-gyn OR ophthalmologist* OR pathologist* OR p#ediatrician* OR psychiatrist* OR oncologist* OR urologist* OR allergist* OR endocrinologist* OR gastroenterologist* OR geriatrician* OR hospitalist* OR nephrologist* OR

pulmonologist* OR rheumatologist* OR clinician* OR nurse* OR midwife* OR midwives) OR ((healthcare OR "health care" OR health OR medical OR nursing) W/3 (professional* OR personnel OR practitioner* OR provider*)))) AND (((TITLE-ABS-KEY (intervention* OR train* OR educat* OR workshop* OR seminar* OR curricul* OR coach* OR mentor* OR experiment* OR tool* OR interview* OR "focus group" OR review* OR screening OR questionnaire* OR survey* OR perception* OR implication* OR barrier* OR facilitat* OR obstacle* OR encourag* OR impediment* OR impede* OR challeng* OR obstruct* OR hurdle OR experience* OR perceive* OR perspective* OR perception* OR "self-report" OR trial* OR test* OR "quality improvement*" OR "quality assurance" OR "patient safety") OR TITLE-ABS-KEY ((qi OR "quality improvement*") W/6 (intervention* OR initiative* OR strateg* OR program* OR campaign* OR implement*)) OR TITLE-ABS-KEY ((practice OR behavio* OR organizational) W/3 chang*))) OR (TITLE-ABS ((((research OR knowledge OR evidence) W/2 (uptake OR "use" OR diffus* OR disseminat* OR utiliz* OR utilis* OR transfer* OR translat* OR implement* OR adopt*)) OR (innovation* W/2 adopt*) OR (innovation* W/2 disseminat*) OR "research into practice" OR "evidence into practice" OR "knowledge to action" OR "know do gap" OR (knowledge W/1 (mobilization OR exchange)) OR "translational science" OR "implementation science"))) OR (TITLE-ABS (guideline* W/2 (introduc* OR issu* OR impact OR effect* OR distribut* OR adher* OR compl* OR utiliz* OR utilis* OR "use" OR uptake OR diffuse* OR transfer* OR implement* OR translat* OR disseminat* OR adopt*))) OR (TITLE-ABS (("evidence informed" W/1 (healthcare OR "health care" OR "decision making")) OR "evidence based practice")))) AND (LIMIT-TO (LANGUAGE , "English"))
Results: 747

Cochrane Library via Wiley
#1 (conscience* or "conscientious object" or "empirical ethics"):ti,ab 133
#2 (doctor* or physician* or "general practitioner*" or GP or surgeon* or anesthesiologist* or cardiologist* or dermatologist* or radiologist* or internist* or geneticist* or neurologist* or obstetrician* or gynecologist* or OBGYN or OB-GYN or ophthalmologist* or pathologist* or pediatrician* or psychiatrist* or oncologist* or urologist* or allergist* or endocrinologist* or gastroenterologist* or geriatrician* or hospitalist* or nephrologist* or pulmonologist* or rheumatologist* or clinician* or nurse* or midwife* or midwives OR ((healthcare or health care or health or medical or nursing) NEAR/3 (professional* or personnel or practitioner* or provider*))):ti,ab 161614
#3 (intervention* or train* or educat* or workshop* or seminar* or curricul* or coach* or mentor* or experiment* or tool* or interview* or focus group or review* or screening or questionnaire* or survey* or perception* or implication* or barrier* or facilitat* or obstacle* or encourag* or impediment* or impede* or challeng* or obstruct* or hurdle or experience* or perceive* or perspective* or perception* or "self-report" or trial* or test* or quality improvement* or "quality assurance" or "patient safety"):ti,ab 1280317
#4 ((QI or "quality improvement") NEAR/6 (intervention* or initiative* or strateg* or program* or campaign* or implement*)):ti,ab 1466
#5 ((Practice or behavio* or organizational) NEAR/3 chang*):ti,ab 14590

#6 ((((research or knowledge or evidence) NEAR/2 (uptake or "use" or diffus* or disseminat* or utiliz* or utilis* or transfer* or translat* or implement* or adopt*)) or (innovation* NEAR/2 adopt*) or (innovation* NEAR/2 disseminat*) or "research into practice" or "evidence into practice" or "knowledge to action" or "know do gap" or (knowledge NEAR/1 (mobilization or exchange)) or "translational science" or "implementation science")):ti,ab 8133
#7 (evidence-informed NEAR/1 (healthcare or health care or decision making)):ti,ab 28
#8 (guideline* NEAR/2 (introduc* or issu* or impact or effect* or distribut* or adher* or compl* or utiliz* or utilis* or "use" or uptake or diffuse* or transfer* or implement* or translat* or disseminat* or adopt*)):ti,ab 2936
#9 evidence based practice:ti,ab 734
#10 {or #3-#9} 1281509
#11 #1 and #2 and #10 43

PhilPapers (no longer available after June 2022)

conscience or "conscientious objection"
AND
health or healthcare or nurse or doctor or physician or midwife or midwives
Limit Pub Date: 2000-2021
Results: 55

Supplementary File 3. MMAT Tool

Category of study designs	Methodological quality criteria	Responses			
		Yes	No	Can't tell	Comments
Screening questions (for all types)	S1. Are there clear research questions?				
	S2. Do the collected data allow to address the research questions?				
	Further appraisal may not be feasible or appropriate when the answer is 'No' or 'Can't tell' to one or both screening questions.				
1. Qualitative	1.1. Is the qualitative approach appropriate to answer the research question?				
	1.2. Are the qualitative data collection methods adequate to address the research question?				
	1.3. Are the findings adequately derived from the data?				
	1.4. Is the interpretation of results sufficiently substantiated by data?				
	1.5. Is there coherence between qualitative data sources, collection, analysis and interpretation?				
2. Quantitative randomized controlled trials	2.1. Is randomization appropriately performed?				
	2.2. Are the groups comparable at baseline?				
	2.3. Are there complete outcome data?				
	2.4. Are outcome assessors blinded to the intervention provided?				
	2.5 Did the participants adhere to the assigned intervention?				
3. Quantitative non-randomized	3.1. Are the participants representative of the target population?				
	3.2. Are measurements appropriate regarding both the outcome and intervention (or exposure)?				
	3.3. Are there complete outcome data?				
	3.4. Are the confounders accounted for in the design and analysis?				
	3.5. During the study period, is the intervention administered (or exposure occurred) as intended?				
4. Quantitative descriptive	4.1. Is the sampling strategy relevant to address the research question?				
	4.2. Is the sample representative of the target population?				
	4.3. Are the measurements appropriate?				
	4.4. Is the risk of nonresponse bias low?				
	4.5. Is the statistical analysis appropriate to answer the research question?				
5. Mixed methods	5.1. Is there an adequate rationale for using a mixed methods design to address the research question?				
	5.2. Are the different components of the study effectively integrated to answer the research question?				
	5.3. Are the outputs of the integration of qualitative and quantitative components adequately interpreted?				
	5.4. Are divergences and inconsistencies between quantitative and qualitative results adequately addressed?				
	5.5. Do the different components of the study adhere to the quality criteria of each tradition of the methods involved?				

Hong QN, Pluye P, Fàbregues S, Bartlett G, Boardman F, Cargo M, Dagenais P, Gagnon M-P, Griffiths F, Nicolau B, O'Cathain A, Rousseau M-C, Vedel I. Mixed Methods Appraisal Tool (MMAT), version 2018. Registration of Copyright (#1148552), Canadian Intellectual Property Office, Industry Canada.

Supplementary File 4. Reasons for exclusion

Study	Reason for exclusion
Ahlin 2013[1]	Wrong study design
Ahlin 2015[2]	Wrong Intervention
Åhlin 2022[3]	Wrong study design
Åhlin 2021[4]	Wrong intervention
Allen 2023[5]	Wrong topic
Backman 2021[6]	Wrong study design
Badro 2011[7]	Wrong study design
Ben-Moshe 2020[8]	Wrong study design
Bentzen 2013[9]	Wrong intervention
Bouthillier 2019[10]	Wrong study design
Bratt 2021[11]	Wrong study design
Braxton 2021[12]	Wrong topic
Brown 2016[13]	Wrong study design
Brown 1996[14]	Wrong study design
Brown 2021[15]	Wrong study design
Cady 2008[16]	Wrong study design
Carnesten 2024[17]	Wrong study design
Catlin 2008[18]	Wrong intervention
Childress 1997[19]	Wrong study design
Ciereszko 2023[20]	Wrong population
Cummins 2021[21]	Wrong study design
Czekajewska 2022[22]	Wrong study design
Dabney 2020[23]	Wrong outcomes
Dahlqvist 2009[24]	Wrong study design
deLondras 2023[25]	Wrong Topic
Dogru 2022[26]	Wrong study design
Duff 2022[27]	Wrong topic
Eble 2021[28]	Wrong intervention
Ericson-Lidman 2014[29]	Wrong topic
Ericson-Lidman 2013a[30]	Wrong intervention
Ericson-Lidman 2018[31]	Wrong study design
Ericson-Lidman 2013b[32]	Wrong study design
Farley 2022[33]	Wrong study design
FischerGronlund 2015[34]	Wrong study design
Fleming 2016[35]	Wrong topic
Fleming 2021[36]	Wrong study design
Fleming 2019[37]	Wrong topic
Ford 2018[38]	Wrong study design
Fovargue 2022[39]	Wrong study design
From 2015[40]	Wrong study design
Gadsby 2021[41]	Wrong study design
Gagné 2023[42]	Wrong study design
Glasberg 2007[43]	Wrong study design
Gorbanzadeh 2016[44]	Wrong study design
Gorbanzadeh 2015[45]	Wrong study design

Grace 2023[46]	Wrong study design
Gulpinar 2021[47]	Wrong population
Gustafsson 2010[48]	Wrong study design
Haining 2021[49]	Wrong intervention
Hanna 2014[50]	Wrong topic
Heikkila 2022[51]	Wrong study design
Heikkila 2021[52]	Wrong intervention
Herttalampi 2023[53]	Wrong study design
Hill 2011[54]	Wrong population
Hrabar 2023[55]	Wrong study design
Jalali 2012[56]	Wrong study design
Jasemi 2019a[57]	Wrong study design
Jasemi 2019b[58]	Wrong study design
Jensen 2009[59]	Wrong study design
Jodaki 2023[60]	Wrong study design
Jodaki 2021[61]	Wrong study design
Jokwiro 2022[62]	Wrong study design
Jokwiro 2021[63]	Wrong study design
Jokwiro 2020[64]	Wrong study design
Jones-Nosacek 2022[65]	Wrong study design
Juthberg 2008[66]	Wrong topic
Juthberg 2007[67]	Wrong study design
Keles 2023[68]	Wrong study design
Khosravani 2017[69]	Wrong study design
Kiska 2018[70]	Wrong intervention
Knox 2023[71]	Wrong study design
Ko 2020[72]	Wrong study design
Koch 2021[73]	Wrong study design
Kono 2023[74]	Wrong topic
Lamb 2021[75]	Wrong study design
Lamb 2019[76]	Wrong study design
Lamb 2019[77]	Wrong study design
Leary 2013[78]	Wrong literature source
Leary 2018[79]	Wrong population
Leonard 2020[80]	Wrong topic
Lewis-Newby 2015[81]	Wrong study design
LimaFontenele 2022[82]	Not in English
Litleskare 2020[83]	Wrong intervention
Logarajah 2016[84]	Wrong study design
LosaIglesias 2010[85]	Wrong intervention
Martins-Vale 2023[86]	Wrong topic
Maxwell 2022[87]	Wrong study design
Mazaheri 2018[88]	Wrong intervention
MICHEL 2020[89]	Wrong intervention
Milionis 2023[90]	Wrong study design
Mohammadi 2020[91]	Wrong intervention
Molin 2020[92]	Wrong outcome
Montero 2022[93]	Wrong study design

Morrison 2023[94]	Wrong study design
Munkeby 2023a[95]	Wrong study design
Munkeby 2023b[96]	Wrong study design
Neill 2021[97]	Wrong population
NgocHuy 2022[98]	Wrong topic
Norberg 2008[99]	Wrong study design
OnerYalcin 2022[100]	Wrong study design
OnerYalcin 2021[101]	Wrong study design
Özcan 2022[102]	Wrong study design
Ozden 2023[103]	Wrong study design
Patthoff 2009[104]	Wrong study design
Puntillo 2001[105]	Wrong intervention
Pusari 1998[106]	Wrong study design
Quintana 2022[107]	Wrong study design
Quintana 2024[108]	Wrong study design
Reichlin 2022[109]	Wrong study design
Saarnio 2012[110]	Wrong intervention
Schrock 1990[111]	Wrong study design
Scott 2022[112]	Wrong study design
Self 2023[113]	Wrong population
Shemie 2017[114]	Wrong intervention
Smith 2009[115]	Wrong study design
Sofaer 1995[116]	Wrong topic
Soriano 2023[117]	Wrong study design
Sorlie 2003[118]	Wrong study design
SutcuCicek 2012[119]	Wrong study design
Symons 2022[120]	Wrong study design
Tai 2006[121]	Wrong study design
Teelin 2022[122]	Wrong topic
Theis 1986[123]	Wrong study design
Tiemersma 2022[124]	Wrong population
Tobin 2022[125]	Wrong study design
Toellner 1994[126]	Wrong topic
Tongue 2022[127]	Wrong topic
Toro-Flores 2019[128]	Wrong study design
Tuvesson 2012[129]	Wrong intervention
VanDiepen 2021[130]	Wrong topic
VanDiepen 2022[131]	Wrong intervention
Varasteh 2022[132]	Wrong topic
West-Oram 2022[133]	Wrong study design
Wilkes 1998[134]	Wrong topic
Williams 2023[135]	Wrong study design
Wronska 1994[136]	Wrong topic
Yang 2023[137]	Wrong study design
Yildirim 2022[138]	Wrong study design
Yildirim 2021[139]	Wrong study design
Zhang 2013[140]	Wrong topic

References

1. Ahlin J, Ericson-Lidman E, Eriksson S, Norberg A, Strandberg G. Longitudinal relationships between stress of conscience and concepts of importance. Nursing ethics 2013;20(8):927–42.

2. Ahlin J, Ericson-Lidman E, Norberg A, Strandberg G. A comparison of assessments and relationships of stress of conscience, perceptions of conscience, burnout and social support between healthcare personnel working at two different organizations for care of older people. Scandinavian journal of caring sciences 2015;29(2):277–87.

3. Åhlin J, Ericson-Lidman E, Strandberg G. Assessments of stress of conscience, burnout and social support amongst care providers in home care and residential care for older people. Scandinavian Journal of Caring Sciences 2022;36(1):131–41.

4. Åhlin J, Ericson-Lidman E, Strandberg G. Assessments of stress of conscience, burnout and social support amongst care providers in home care and residential care for older people. Scandinavian Journal of Caring Sciences 2021;1.

5. Allen D. What is moral injury and how does it affect nurses?: When healthcare staff have to make care decisions that go against their values, a conflict of conscience can arise. Emergency Nurse 2023;31(3):10–1.

6. Backman A, Sjögren K, Lövheim H, Lindkvist M, Edvardsson D. The influence of nursing home managers' leadership on person-centred care and stress of conscience: A cross-sectional study. BMC Nursing 2021;20(1):1–8.

7. Badro V. Dilemmas of conscience in the practice of medicine: a phenomenological study. Narrative inquiry in bioethics 2011;1(3):171–88.

8. Ben-Moshe N. Conscientious Objection in Medicine: Making it Public. HEC forum□: an interdisciplinary journal on hospitals' ethical and legal issues 2020;(aha, 8917455).

9. Bentzen G, Harsvik A, Brinchmann BS. "Values that vanish into thin air": nurses' experience of ethical values in their daily work. Nursing research and practice 2013;2013(101561211):939153.

10. Bouthillier M-E, Opatrny L. A qualitative study of physicians' conscientious objections to medical aid in dying. Palliative medicine 2019;33(9):1212–20.

11. Bratt AS, Johansson M, Holmberg M, et al. An internet-based compassion course for healthcare professionals: Rationale and protocol for a randomised controlled trial. Internet Interventions [Internet] 2021;Available from: https://www.scopus.com/inward/record.uri?eid=2-s2.0-85116622972&doi=10.1016%2fj.invent.2021.100463&partnerID=40&md5=393d9b941dc2687c0c033dd7d0633eff

12. Braxton JM, Busse EM, Rushton CH. Mapping the Terrain of Moral Suffering. Perspectives in biology and medicine 2021;64(2):235–45.

13. Brown BP, Hasselbacher L, Chor J. Whose Choice? Developing a Unifying Ethical Framework for Conscience Laws in Health Care. Obstetrics and gynecology 2016;128(2):391–5.

14. Brown JM. Conscience: the professional and the personal. Journal of nursing management 1996;4(3):171–7.

15. Brown J, Goodridge D, Thorpe L, Hodson A, Chipanshi M. Factors influencing practitioners' who do not participate in ethically complex, legally available care: scoping review. BMC medical ethics 2021;22(1):134.

16. Cady R.F. Refusal to care. JONA'S healthcare law, ethics and regulation 2008;10(2).

17. Carnesten H., von Heideken Wagert P., Gustin L.W., et al. Struggling in the dehumanized world of COVID-An exploratory mixed-methods study of frontline healthcare workers' experiences. Journal of advanced nursing 2024;((Carnesten, von Heideken Wagert, Gustin, Toivanen, Skoglund, Andreae) School of Health, Care and Social Welfare, Malardalen University, Eskilstuna/Vasteras, Sweden(Gustin) Department of Health and Care Sciences, UiT/The Arctic University of Norway, Narvik).

18. Catlin A, Armigo C, Volat D, et al. Conscientious objection: a potential neonatal nursing response to care orders that cause suffering at the end of life? Study of a concept. Neonatal network□: NN 2008;27(2):101–8.

19. Childress JF. Conscience and conscientious actions in the context of MCOs. Kennedy Institute of Ethics journal 1997;7(4):403–11.

20. Ciereszko K, Napiwodzka K, Nowak E, Hemmerling K. Women's Reproductive Health Rights in Poland. Between a Druggists' Conscience Clause and Their Legal Duty to Provide Contraceptives. Ethics in Progress 2023;14(1):94–109.

21. Cummins PJ. Conscientious Objection and Physician-Employees. HEC forum□: an interdisciplinary journal on hospitals' ethical and legal issues 2021;33(3):247–68.

22. Czekajewska J, Walkowiak D, Domaradzki J. Attitudes of Polish physicians, nurses and pharmacists towards the ethical and legal aspects of the conscience clause. BMC medical ethics 2022;23(1):107.

23. Dabney C, Appling NA, Herr MJ. An Interprofessional Branching Simulation to Introduce RN First Assistant Students to Their Role in the Perioperative Setting. AORN journal 2020;112(5):471–7.

24. Dahlqvist V, Soderberg A, Norberg A. Facing inadequacy and being good enough: Psychiatric care providers' narratives about experiencing and coping with troubled conscience. Journal of Psychiatric and Mental Health Nursing 2009;16(3):242–7.

25. de Londras F, Cleeve A, Rodriguez MI, Farrell A, Furgalska M, Lavelanet AF. The Impact of “conscientious objection” on abortion-related outcomes: A synthesis of legal and health evidence. Health policy (Amsterdam, Netherlands) 2023;129(8409431, hep):104716.

26. Dogru BV, Utli H. Determining the Correlation between Intercultural Sensitivity and Compassion and Conscience Levels of Internal Medicine Clinical Nurses in the South-East of Turkey. International Journal of Caring Sciences 2022;15(2):893–904.

27. Duff J, Bowen L, Gumuskaya O. What does surgical conscience mean to perioperative nurses: An interpretive description. Collegian 2022;29(2):147–53.

28. Eble J. Implications of John Kavanaugh’s Philosophy of the Human Person as Embodied Reflexive Consciousness for Conscientious Decision-making in Brain Death. The Linacre quarterly 2021;88(1):71–81.

29. Ericson-Lidman E, Larsson L-LF, Norberg A. Caring for people with dementia disease (DD) and working in a private not-for-profit residential care facility for people with DD. Scandinavian journal of caring sciences 2014;28(2):337–46.

30. Ericson-Lidman E, Strandberg G. Dealing with troubled conscience in municipal care of older people. Nursing ethics 2013;20(3):300–11.

31. Ericson-Lidman E, Strandberg G. Using a developed participatory action research process in practice to help care providers deal with troubled conscience in residential care of older people. Action Research 2018;16(2):190–206.

32. Ericson-Lidman E, Norberg A, Persson B, Strandberg G. Healthcare personnel’s experiences of situations in municipal elderly care that generate troubled conscience. Scandinavian journal of caring sciences 2013;27(2):215–23.

33. Farley M. SURGICAL CONSCIENCE AND ITS ROLE IN PATIENT SAFETY AND CARE. ORNAC Journal 2022;40(1):15–30.

34. Fischer Gronlund CEC, Soderberg AIS, Zingmark KM, Sandlund SM, Dahlqvist V. Ethically difficult situations in hemodialysis care - Nurses’ narratives. Nursing ethics 2015;22(6):711–22.

35. Fleming KA, Heintzelman SJ, Bartholow BD. Specifying Associations Between Conscientiousness and Executive Functioning: Mental Set Shifting, Not Prepotent Response Inhibition or Working Memory Updating. Journal of personality 2016;84(3):348–60.

36. Fleming V, Frith L, Ramsayer B. Tensions Between Ethics and the Law: Examination of a Legal Case by Two Midwives Invoking a Conscientious Objection to Abortion in Scotland. HEC forum□: an interdisciplinary journal on hospitals’ ethical and legal issues 2021;33(3):189–213.

37. Fleming V, Robb Y. Potential conflicts in midwifery practice regarding conscientious objection to abortions in Scotland. Nursing ethics 2019;26(2):564–75.

38. Ford NJ, Austin W. Conflicts of conscience in the neonatal intensive care unit: Perspectives of Alberta. Nursing ethics 2018;25(8):992–1003.

39. Fovargue S, Neal M. UK pharmacists' experiences and perceptions of conflict between personal ethical commitments and professional obligations, as set out in professional guidance. The International journal of pharmacy practice 2022;30(3):241–6.

40. From I, Wilde-Larsson B, Nordstrom G, Johansson I. Formal caregivers' perceptions of quality of care for older people: associating factors. BMC research notes 2015;8(101462768):623.

41. Gadsby J, McKeown M. Mental health nursing and conscientious objection to forced pharmaceutical intervention. Nursing philosophy□: an international journal for healthcare professionals 2021;22(4).

42. Gagné M-A. Factors influencing the involvement of health care professionals in the administration of medical aid in dying in Quebec. Ethics, Medicine and Public Health [Internet] 2023;29. Available from: https://www.scopus.com/inward/record.uri?eid=2-s2.0-85160253272&doi=10.1016%2fj.jemep.2023.100911&partnerID=40&md5=d3db11a7add9881b926233eac68c1781

43. Glasberg AL, Eriksson S, Norberg A. Burnout and "stress of conscience" among healthcare personnel. Journal of advanced nursing 2007;57(4):392–403.

44. Gorbanzadeh B, Rahmani A, Mogadassian S, Behshid M, Azadi A, Taghavy S. Levels of conscience and related factors among Iranian oncology nurses. Asian Pacific Journal of Cancer Prevention 2016;16(18):8211–4.

45. Gorbanzadeh B, Rahmani A, Mogadassian S, Behshid M, Azadi A, Taghavy S. Levels of Conscience and Related Factors among Iranian Oncology Nurses. Asian Pacific journal of cancer prevention□: APJCP 2015;16(18):8211–4.

46. Grace PJ, Peter E, Lachman VD, Johnson NL, Kenny DJ, Wocial LD. Professional responsibility, nurses, and conscientious objection: A framework for ethical evaluation. Nursing ethics 2023;(b6m, 9433357):9697330231180749.

47. Gulpinar G, Keles S, Yalim NY. Perspectives of community pharmacists on conscientious objection to provide pharmacy services: A theory informed qualitative study. Journal of the American Pharmacists Association□: JAPhA 2021;61(4):373-381.e1.

48. Gustafsson G, Eriksson S, Strandberg G, Norberg A. Burnout and perceptions of conscience among health care personnel: a pilot study. Nursing ethics 2010;17(1):23–38.

49. Haining CM, Keogh LA, Gillam LH. Understanding the Reasons Behind Healthcare Providers' Conscientious Objection to Voluntary Assisted Dying in Victoria, Australia. Journal of bioethical inquiry 2021;(101250741).

50. Hanna T, Mona E. Psychosocial work environment, stress factors and individual characteristics among nursing staff in psychiatric in-patient care. International journal of environmental research and public health 2014;11(1):1161–75.

51. Heikkila M, Huhtala M, Mauno S, Feldt T. Intensified job demands, stress of conscience and nurses' experiences during organizational change. Nursing ethics 2022;29(1):217–30.

52. Heikkila M, Huhtala M, Mauno S, Feldt T. Intensified job demands, stress of conscience and nurses' experiences during organizational change. Nursing ethics 2021;(b6m, 9433357):9697330211006831.

53. Herttalampi M, Feldt T. A new approach to stress of conscience's dimensionality: Hindrance and violation stressors and their role in experiencing burnout and turnover intentions in healthcare. Journal of clinical nursing 2023;(bzz, 9207302).

54. Hill PL, Roberts BW. The Role of Adherence in the Relationship Between Conscientiousness and Perceived Health. Health Psychology 2011;30(6):797–804.

55. Hrabar D. The Conscientious Objection of Physicians (Gynecologists) and the Value System. Donald School Journal of Ultrasound in Obstetrics and Gynecology 2023;17(1):36–46.

56. Jalali R, Hasani P, Abedsaeedi AZ. Nurses' Experience of the Perception of Nursing Conscience: A Phenomenological Study. Asian Bioethics Review 2012;4(3):210–25.

57. Jasemi M, Purteimor S, Zabihi RE, Pak MHM, Eghtedar S. Nurses' Strategies for Conscience-based Care Delivery: A Qualitative Study. Indian journal of palliative care 2019;25(4):517–22.

58. Jasemi M, Aazami S, Hemmati Maslak Pak M, Habibzadeh H, Esmaeili Zabihi R. Factors affecting conscience-based nursing practices: A qualitative study. Nursing ethics 2019;26(5):1350–60.

59. Jensen A, Lidell E. The influence of conscience in nursing. Nursing ethics 2009;16(1):31–42.

60. Jodaki K, Esmaeili M, Cheraghi MA, Mazaheri M. Striving to Keep a Clear Conscience by Going Above and Beyond: The Experiences of Intensive Care Unit Nurses. Critical care nursing quarterly 2023;46(2):192–202.

61. Jodaki K, Esmaeili M, Cheraghi MA, Pashaeypoor S, Sadat Hoseini AS. Clarifying the concept of conscience in nurses' ethical performance in Iran: a

concept analysis study. Journal of medical ethics and history of medicine 2021;14(101606442):14.

62. Jokwiro Y., Wilson E., Bish M. The extent and nature of stress of conscience among healthcare workers: A scoping review. Applied nursing research□: ANR 2022;63((Jokwiro, Bish) Department of Rural Nursing&Midwifery, La Trobe Rural Health School, Australia(Wilson) Rural Dept of Community Health, La Trobe Rural Health School, Australia):151554.

63. Jokwiro Y, Rickard N, Edvardsson D. Factors associated with stress of conscience in caring for older people with delirium in a hospital setting: An exploratory cross-sectional study. Collegian 2021;28(3):296–302.

64. Jokwiro Y, Pascoe E, Edvardsson K, et al. Stress of Conscience Questionnaire (SCQ): exploring dimensionality and psychometric properties at a tertiary hospital in Australia. BMC psychology 2020;8(1):109.

65. Jones-Nosacek C. Referral vs Transfer of Care: Ethical Options When Values Differ. The Linacre quarterly 2022;89(1):36–46.

66. Juthberg C, Eriksson S, Norberg A, Sundin K. Stress of conscience and perceptions of conscience in relation to burnout among care-providers in older people. Journal of clinical nursing 2008;17(14):1897–906.

67. Juthberg C, Eriksson S, Norberg A, Sundin K. Perceptions of conscience in relation to stress of conscience. Nursing ethics 2007;14(3):329–43.

68. Keles S, Dag O, Aksu M, Gulpinar G, Yalim NY. Development of the Inclination Toward Conscientious Objection Scale for Physicians. Health care analysis□: HCA□: journal of health philosophy and policy 2023;31(2):81–98.

69. Khosravani M, Abedi HA, Lak S, Rafiei F, Rahzani K. The association between conscience understanding and clinical performance among nurses working at education hospital of Arak. Annals of Tropical Medicine and Public Health 2017;10(6):1587–90.

70. Kiska R. Freedom of conscience: a benefit to health care worker and patient alike. International Journal of Human Rights in Healthcare 2018;11(2):144–52.

71. Knox M, Wagg A. Contemplating the Impacts of Canadian Healthcare Institutions That Refuse to Provide Medical Assistance in Dying: A Framework-Based Discussion of Potential Health Access Implications. The American journal of hospice & palliative care 2023;(9008229, atd):10499091231155854.

72. Ko CM, Koh CK, Lee YS. An ethical issue: nurses’ conscientious objection regarding induced abortion in South Korea. BMC medical ethics 2020;21(1):106.

73. Koch T. The practitioner as endangered citizen: a genealogy. Monash bioethics review 2021;39(2):157–68.

74. Kono M, Arai N, Takimoto Y. Identifying practical clinical problems in active euthanasia: A systematic literature review of the findings in countries where euthanasia is legal. Palliative & supportive care 2023;21(4):705–13.

75. Lamb C. Conscience: an investigation in stenian philosophy in relation to bioethics. Religions [Internet] 2021;12(8). Available from: https://www.scopus.com/inward/record.uri?eid=2-s2.0-85111589470&doi=10.3390%2frel12080566&partnerID=40&md5=0899262e87a32bc38dbcbe4141e8b63c

76. Lamb C, Evans M, Babenko-Mould Y, Wong C, Kirkwood K. Nurses' use of conscientious objection and the implications for conscience. Journal of advanced nursing 2019;75(3):594–602.

77. Lamb C, Babenko-Mould Y, Evans M, Wong CA, Kirkwood KW. Conscientious objection and nurses: Results of an interpretive phenomenological study. Nursing ethics 2019;26(5):1337–49.

78. Leary S. Relationship of internet-delivered Mantram Repetition Program to burnout, stress of conscience, spiritual wellbeing, and mindfulness in healthcare workers. University of San Diego; 2013.

79. Leary S, Weingart K, Topp R, Bormann J. The Effect of Mantram Repetition on Burnout and Stress Among VA Staff. Workplace Health & Safety 2018;66(3):120–8.

80. Leonard G, Cao J, Scielzo S, et al. The Effect of Stress and Conscientiousness on Simulated Surgical Performance in Unbalanced Groups: a Bayesian Hierarchical Model. 2020;231(4):S258-.

81. Lewis-Newby M, Wicclair M, Pope T, et al. An official American Thoracic Society policy statement: managing conscientious objections in intensive care medicine. American journal of respiratory and critical care medicine 2015;191(2):219–27.

82. Lima Fontenele A, de Souza Paes Landim J, de Souza Domingues PH, et al. CONSCIENTIOUS OBJECTION AND ABORTION: OPINIONS AND KNOWLEDGE OF NURSING STUDENTS. Ciencia, Cuidado e Saude 2022;21:1–9.

83. Litleskare LA, Strander MT, Forde R, Magelssen M. Refusals to perform ritual circumcision: a qualitative study of doctors' professional and ethical reasoning. BMC medical ethics 2020;21(1):5.

84. Logarajah S, Roff S. Structured learning for clinical ethics in anaesthesia. Clinical Ethics 2016;11(4):200–9.

85. Losa Iglesias ME, Becerro de Bengoa Vallejo R, Salvadores Fuentes P. Moral distress related to ethical dilemmas among Spanish podiatrists. Journal of medical ethics 2010;36(5):310–4.

86. Martins-Vale M, Pereira HP, Marina S, Ricou M. Conscientious Objection and Other Motivations for Refusal to Treat in Hastened Death: A Systematic Review. Healthcare (Basel, Switzerland) 2023;11(15).

87. Maxwell C, Ramsayer B, Fleming V. It’s about finding a balance...exploring conscientious objection to abortion with UK midwives. Midwifery 2022;112(8510930, mwf):103416.

88. Mazaheri M, Ericson-Lidman E, Joakim O, Norberg A. Meanings of troubled conscience and how to deal with it: expressions of Persian-speaking enrolled nurses in Sweden. Scandinavian journal of caring sciences 2018;32(1):380–8.

89. MICHEL AR, KUNG S, LÓPEZ-SALM A, NAVARRETE SA. Regulating Conscientious Objection to Legal Abortion in Argentina: Taking into Consideration Its Uses and Consequences. Health & Human Rights: An International Journal 2020;22(2):271–83.

90. Milionis C, Toska A. Conscientious objection to caring for transgender people: An ethical right or a discriminatory attitude?. International journal of nursing practice 2023;(CVU, 9613615):e13180.

91. Mohammadi A, Hanifi N, Varjoshani NJ. The relationship amongst nurses’ perceived organizational justice, work consciousness, and responsibility. Nursing ethics 2020;27(3):701–13.

92. Molin J, Hällgren Graneheim U, Ringnér A, Lindgren B. Time Together as an arena for mental health nursing–staff experiences of introducing and participating in a nursing intervention in psychiatric inpatient care. International Journal of Mental Health Nursing 2020;29(6):1192–201.

93. Montero A, Ramirez-Pereira M, Robledo P, Casas L, Vivaldi L, Gonzalez D. Conscientious objection as structural violence in the voluntary termination of pregnancy in Chile. Frontiers in psychology 2022;13(101550902):1007025.

94. Morrison SD, Nolan IT, Santosa K, Shuman AG, Vercler CJ, Kuzon WMJ. Conscientious Objection to Gender-Affirming Surgery: Institutional Experience and Recommendations. Plastic and reconstructive surgery 2023;152(1):217–20.

95. Munkeby H, Bratberg G, Devik SA. Registered nurses’ exposure to high stress of conscience in long-term care. Nursing ethics 2023;(b6m, 9433357):9697330231167542.

96. Munkeby H, Bratberg G, Devik SA. Meanings of troubled conscience in nursing homes: nurses’ lived experience. Nursing ethics 2023;30(1):20–31.

97. Neill MS. Public Relations Professionals Identify Ethical Issues, Essential Competencies and Deficiencies. Journal of Media Ethics 2021;36(1):51–67.

98. Ngoc Huy DT, Khalil NR, Le K, Mahdi AB, Djuraeva L. Religious beliefs and work conscience of Muslim nurses in Iraq during the COVID-19 pandemic. Hervormde Teologiese Studies 2022;78(4):1–6.

99. Norberg A, Eriksson S, Glasberg A-L. Factors associated with “stress of conscience” in healthcare. Scandinavian Journal of Caring Sciences 2008;22(2):249–58.

100. Oner Yalcin S, Yildirim G, Kadioglu FG, Sertdemir Y. Stress of conscience and burnout among nurses in Turkey. Perspectives in psychiatric care 2022;58(4):1882–90.

101. Oner Yalcin S, Yildirim G, Kadioglu FG, Sertdemir Y. Stress of conscience and burnout among nurses in Turkey. Perspectives in psychiatric care 2021;(ozt, 0401133).

102. Özcan Ş, Yanmış S. Have we been able to improve the conscientious intelligence levels of nursing students sufficiently? Perspectives in Psychiatric Care 2022;58(4):1873–81.

103. Ozden G, Parlar Kilic S. Compassion in action: Exploring the relationship between nurse conscientious intelligence and palliative care. Nursing in critical care 2023;(9808649, c3k).

104. Patthoff D, Corsino BV. Acceptance, universal patient acceptance, and access to care an update. Linacre Quarterly 2009;76(1):47–67.

105. Puntillo KA, Benner P, Drought T, et al. End-of-life issues in intensive care units: a national random survey of nurses’ knowledge and beliefs. American journal of critical care: an official publication, American Association of Critical-Care Nurses 2001;10(4):216–29.

106. Pusari ND. Eight “Cs” of caring: a holistic framework for nursing terminally ill patients. Contemporary nurse 1998;7(3):156–60.

107. Quintana D. Surgical Conscience: A Concept Analysis for Perioperative Nurses. AORN journal 2022;116(6):533–46.

108. Quintana D, Keele R, Fredland N, Woo J. Development and Validation of an Instrument to Measure Barriers to Surgical Conscience Action in Perioperative Nurses. Journal of nursing measurement 2024;(b6l, 9318902).

109. Reichlin M. The Reasonableness Standard for Conscientious Objection in Healthcare. Journal of Bioethical Inquiry 2022;19(2):255–64.

110. Saarnio R, Sarvimaki A, Laukkala H, Isola A. Stress of conscience among staff caring for older persons in Finland. Nursing ethics 2012;19(1):104–15.

111. Schrock RA. Conscience and courage--a critical examination of professional conduct. Nurse education today 1990;10(1):3–9.

112. Scott D.C. Democratizing Conscientious Refusal in Healthcare. HEC forum: an interdisciplinary journal on hospitals’ ethical and legal issues 2022;((Scott) Bellarmine University, 2001 Newburg Rd, Louisville, KY 40205, United States).

113. Self B, Maxwell C, Fleming V. The missing voices in the conscientious objection debate: British service users' experiences of conscientious objection to abortion. BMC medical ethics 2023;24(1):65.

114. Shemie SD, Simpson C, Blackmer J, et al. Ethics Guide Recommendations for Organ-Donation-Focused Physicians: Endorsed by the Canadian Medical Association. Transplantation 2017;101(5S Suppl 1):S41–7.

115. Smith WJ. Defending the Hippocratic Oath: The Importance of Conscience in Health Care. Human Life Review 2009;35(1/2):63–70.

116. Sofaer B. Enhancing humanistic skills: an experiential approach to learning about ethical issues in health care. Journal of medical ethics 1995;21(1):31–4.

117. Soriano V, Montero B. Current Challenges for Conscientious Objection by Physicians in Spain. Linacre Quarterly [Internet] 2023;Available from: https://www.scopus.com/inward/record.uri?eid=2-s2.0-85167794238&doi=10.1177%2f00243639231184352&partnerID=40&md5=702850b51d8ba93c23d249c2e2a95cb9

118. Sorlie V, Jansson L, Norberg A. The meaning of being in ethically difficult care situations in paediatric care as narrated by female Registered Nurses. Scandinavian journal of caring sciences 2003;17(3):285–92.

119. Sutcu Cicek H, Demirtas A, Tosun N, Akbayrak N, Yava A, Ozcan CT. The experiences of intensive care nurses caring for patients in vegetative state. Anatolian Journal of Clinical Investigation 2012;6(3):162–8.

120. Symons X. Conscientious Objection in Health Care: Why the Professional Duty Argument is Unconvincing. The Journal of medicine and philosophy 2022;47(4):549–57.

121. Tai M.C.-T., Hsin D.H.-C. Value neutrality and a bioethics of conscience. Tzu Chi Medical Journal 2006;18(1):69–73.

122. Teelin KL, Shubkin CD, Caruso Brown AE. Conscientious Objection to Providing Gender Health Care in Pediatric Training: Balancing the Vulnerability of Transgender Youth and the Vulnerability of Pediatric Residents. The Journal of pediatrics 2022;240(jlz, 0375410):272–9.

123. Theis EC. Ethical issues: A nursing perspective. The New England Journal of Medicine 1986;315(19):1222–4.

124. Tiemersma J., Noom M., Stoffelsen R., Popma A., Schalkwijk F.S. Conceptualisation and conscience assessment in adolescence in forensic mental healthcare: experts' perspectives. Journal of Forensic Psychiatry and Psychology 2022;33(5):673–91.

125. Tobin B. Respect for conscientious judgement in health care. Journal of Paediatrics & Child Health 2022;58(10):1729–30.

126. Toellner R. Experiences with committees dealing with the issues of ethics. Forensic Science International 1994;69(3):329–36.

127. Tongue Z.L. On conscientious objection to abortion: Questioning mandatory referral as compromise in the international human rights framework. Medical Law International 2022;22(4):349–71.

128. Toro-Flores R, Bravo-Agui P, Catalan-Gomez MV, et al. Opinions of nurses regarding conscientious objection. Nursing ethics 2019;26(4):1027–38.

129. Tuvesson H, Eklund M, Wann-Hansson C. Stress of Conscience among psychiatric nursing staff in relation to environmental and individual factors. Nursing ethics 2012;19(2):208–19.

130. Van Diepen C., Fors A., Ekman I., Bertilsson M., Hensing G. Associations between person-centred care and job strain, stress of conscience, and intent to leave among hospital personnel. Journal of clinical nursing 2021;((Van Diepen, Fors, Ekman) Institute of Health and Care Sciences, Sahlgrenska Academy, University of Gothenburg, Gothenburg, Sweden(Van Diepen) Erasmus School of Health Policy&Management, Erasmus University Rotterdam, Rotterdam, Netherlands(Van Diepen, F).

131. Van Diepen C, Fors A, Ekman I, Bertilsson M, Hensing G. Associations between person-centred care and job strain, stress of conscience, and intent to leave among hospital personnel. Journal of clinical nursing 2022;31(5–6):612–22.

132. Varasteh S, Esmaeili M, Mazaheri M. Factors affecting Iranian nurses' intention to leave or stay in the profession during the COVID-19 pandemic. International nursing review 2022;69(2):139–49.

133. West-Oram PGN, Nunes JAA. Conscience absolutism via legislative amendment. Clinical Ethics 2022;17(3):225–9.

134. Wilkes LM, Wallis MC. A model of professional nurse caring: nursing students' experience. Journal of advanced nursing 1998;27(3):582–9.

135. Williams A. Understanding Conscientious Objection and the Acceptability of its Practice in Primary Care. The New bioethics□: a multidisciplinary journal of biotechnology and the body 2023;29(2):156–80.

136. Wronska I, Baajer M. Is moral education of nurses still possible? Nursing Ethics 1994;1(3):131–5.

137. Yang Q, Zheng Z, Pang S, et al. Clinical nurse adherence to professional ethics: A grounded theory. Nursing ethics 2023;30(2):197–209.

138. Yildirim G, Kaya N, Altunbas N. Relationship between nurses' perceptions of conscience and perceptions of individualized nursing care: A cross-sectional study. Perspectives in psychiatric care 2022;58(4):1564–75.

139. Yildirim G, Kaya N, Altunbas N. Relationship between nurses' perceptions of conscience and perceptions of individualized nursing care: A cross-sectional study. Perspectives in psychiatric care 2021;(ozt, 0401133).

140. Zhang H, Liu Y, Wang K, Chan DK-S. Factors associated with stress of conscience among emergency medical technicians in China. International Journal of Nursing Practice 2013;19(SUPPL.3):89–96.
